# Biosynthesis of the Stress-Protectant and Chemical Chaperon Ectoine: Biochemistry of the Transaminase EctB

**DOI:** 10.3389/fmicb.2019.02811

**Published:** 2019-12-10

**Authors:** Alexandra A. Richter, Christopher-Nils Mais, Laura Czech, Kyra Geyer, Astrid Hoeppner, Sander H. J. Smits, Tobias J. Erb, Gert Bange, Erhard Bremer

**Affiliations:** ^1^Laboratory for Microbiology, Department of Biology, Philipps-University Marburg, Marburg, Germany; ^2^SYNMIKRO Research Center, Philipps-University Marburg, Marburg, Germany; ^3^Department of Chemistry, Philipps-University Marburg, Marburg, Germany; ^4^Department of Biochemistry and Synthetic Metabolism, Max Planck Institute for Terrestrial Microbiology, Marburg, Germany; ^5^Center for Structural Studies, Heinrich Heine University Düsseldorf, Düsseldorf, Germany; ^6^Institute of Biochemistry, Heinrich Heine University Düsseldorf, Düsseldorf, Germany

**Keywords:** osmotic stress, compatible solutes, transaminase, pyridoxal-5′-phosphate, ABC transporters

## Abstract

Bacteria frequently adapt to high osmolarity surroundings through the accumulation of compatible solutes. Ectoine is a prominent member of these types of stress protectants and is produced via an evolutionarily conserved biosynthetic pathway beginning with the L-2,4-diaminobutyrate (DAB) transaminase (TA) EctB. Here, we studied EctB from the thermo-tolerant Gram-positive bacterium *Paenibacillus lautus* (*Pl*) and show that this tetrameric enzyme is highly tolerant to salt, pH, and temperature. During ectoine biosynthesis, EctB converts L-glutamate and L-aspartate-beta-semialdehyde into 2-oxoglutarate and DAB, but it also catalyzes the reverse reaction. Our analysis unravels that EctB enzymes are mechanistically identical to the PLP-dependent gamma-aminobutyrate TAs (GABA-TAs) and only differ with respect to substrate binding. Inspection of the genomic context of the *ectB* gene in *P. lautus* identifies an unusual arrangement of juxtapositioned genes for ectoine biosynthesis and import via an Ehu-type binding-protein-dependent ABC transporter. This operon-like structure suggests the operation of a highly coordinated system for ectoine synthesis and import to maintain physiologically adequate cellular ectoine pools under osmotic stress conditions in a resource-efficient manner. Taken together, our study provides an in-depth mechanistic and physiological description of EctB, the first enzyme of the ectoine biosynthetic pathway.

## Introduction

Fluctuations in the environmental osmolarity are frequently encountered challenges that most free-living microorganisms have to cope with in their ecophysiologically varied habitats (Galinski and Trüper, 1994; Kempf and Bremer, 1998; Wood et al., 2001; Gunde-Cimerman et al., 2018). As a consequence of the considerable osmotic potential of the cytoplasm, increases and decreases in the external osmolarity inevitably trigger water fluxes across the semipermeable cytoplasmic membrane (Wood, 2011; Bremer and Krämer, 2019). These accelerated water fluxes negatively impinge on key cellular processes. At high external osmolarity, water efflux prevents the proper hydration of the cytoplasm and decreases the magnitude of vital turgor to physiologically inappropriate values (Wood, 2011; Rojas and Huang, 2017). When the external osmolarity suddenly drops, increased water influx can affect cellular integrity as the result of an excessive increase in turgor (Booth, 2014; Cox et al., 2018). To avert these detrimental effects, microorganisms actively modulate the osmotic potential of their crowded cytoplasm in order to indirectly guide and scale compensatory water fluxes (Wood, 2011; van den Berg et al., 2017; Bremer and Krämer, 2019).

When faced with high osmolarity surroundings, many bacteria initially accumulate potassium as an emergency stress reaction and subsequently replace most of this ion with a special group of organic compounds, the so-called compatible solutes (Csonka, 1989; Galinski and Trüper, 1994; Kempf and Bremer, 1998; Wood, 1999; Wood et al., 2001). Combined, these adaptive responses promote hydration of the cytoplasm and adjustment of turgor to values conducive for growth under osmotically challenging conditions without generating a long-lasting high-ionic-strength cytoplasm (Csonka, 1989; Galinski and Trüper, 1994; Kempf and Bremer, 1998; Wood, 1999; Gunde-Cimerman et al., 2018). Compatible solutes are a chemically diverse group of highly water-soluble organic osmolytes (da Costa et al., 1998; Roberts, 2004) that are compliant with cellular biochemistry and physiology (Bolen and Baskakov, 2001; Rani and Venkatesu, 2018). Building on their physico-chemical attributes, compatible solutes can be accumulated to exceedingly high levels, and the degree of the imposed osmotic stress determines the size of their cellular pools (Csonka, 1989; Kempf and Bremer, 1998; Wood, 1999; Wood et al., 2001). In addition to their role in osmoregulation, they can also act as chemical chaperones (Diamant et al., 2001; Chattopadhyay et al., 2004) by promoting the functionality of key cellular processes under intracellular unfavorable conditions (Lippert and Galinski, 1992; Barth et al., 2000; Bourot et al., 2000; Yancey, 2004; Ignatova and Gierasch, 2006; Stadmiller et al., 2017).

**FIGURE 1:**
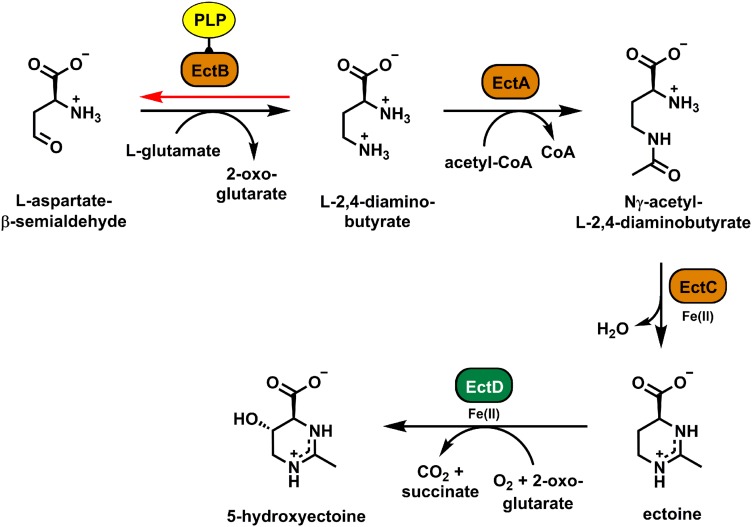
Biosynthetic pathway for ectoine and hydroxyectoine. The data specifying the activities of the ectoine (EctABC) and hydroxyectoine (EctD) biosynthetic enzymes were compiled from the literature (Peters et al., 1990; Ono et al., 1999; Bursy et al., 2007; Pastor et al., 2010; Reshetnikov et al., 2011; Czech et al., 2018a). EctB catalyzes the first step of ectoine biosynthesis; it mediates the PLP-dependent reversible conversion of L-aspartate-β-semialdehyde and L-glutamate into 2-oxoglutarate and L-2,4-diaminobutyrate (DAB) (black arrow). The red arrow indicates the “reverse reaction” of the EctB enzyme (red), which is also dependent on the cofactor PLP (yellow), and which was used in this study to assess the biochemical activities of the (*Pl*)EctB aminotransferase.

Ectoine [(*S*)-2-methyl-1,4,5,6-tetrahydropyrimidine-4-carbo- xylic acid] (Galinski et al., 1985) and its derivative 5-hydroxyectoine [(4*S*,5*S*)-5-hydroxy-2-methyl-1,4,5,6-tetrahyd- ropyrimidine-4-carboxylic acid] (Inbar and Lapidot, 1988; Figure 1) are prominent members of the compatible solutes. They are widely used as stress protectants by microorganisms (da Costa et al., 1998; Pastor et al., 2010; Kunte et al., 2014; Czech et al., 2018a; Gunde-Cimerman et al., 2018). Synthesis of ectoines provides a considerable degree of osmotic stress tolerance (Galinski and Trüper, 1994; Pastor et al., 2010; Kunte et al., 2014; Czech et al., 2018a), can confer protection against extremes in both low or high growth temperatures (Garcia-Estepa et al., 2006; Bursy et al., 2008; Kuhlmann et al., 2008; Salvador et al., 2018), can ameliorate desiccation stress (Manzanera et al., 2004; Tanne et al., 2014), can protect the functionality of proteins against various types of challenges (Lippert and Galinski, 1992; Knapp et al., 1999; Barth et al., 2000; Kolp et al., 2006), can stabilize lipid bilayers (Harishchandra et al., 2010), can protect DNA from damage by ionizing radiation (Schröter et al., 2017), and provides hydroxyl radical scavenging activity (Brands et al., 2019). The function-preserving attributes of ectoines for proteins, cell membranes, DNA, and macromolecular complexes attracted considerable biotechnological attention (Lentzen and Schwarz, 2006; Graf et al., 2008; Kurz, 2008; Pastor et al., 2010; Kunte et al., 2014; Czech et al., 2018a). This led to the development of an industrial-scale production process using the salt-tolerant bacterium *Halomonas elongata* as the production host (Pastor et al., 2010; Schwibbert et al., 2011; Kunte et al., 2014). Ectoines are commercially high-value compounds, and consequently, there are now continued efforts to improve the productivity of both natural (Schiraldi et al., 2006; Pastor et al., 2010; Rodriguez-Moya et al., 2013; Kunte et al., 2014; Cantera et al., 2018; Chen et al., 2018) and synthetic cell factories (Seip et al., 2011; Rodriguez-Moya et al., 2013; Chen et al., 2015; Czech et al., 2018b; Giesselmann et al., 2019). Hence, both from the perspective of basic science and applied approaches, a detailed understanding of the ectoine biosynthetic enzymes are highly desirable.

Biosynthesis of ectoine (Figure 1) initiates from the central microbial metabolite L-aspartate-β-semialdehyde (L-ASA) (Peters et al., 1990; Reshetnikov et al., 2006; Lo et al., 2009; Reshetnikov et al., 2011; Stöveken et al., 2011). It is mediated by three enzymes: L-2,4-diaminobutyrate (DAB) transaminase (TA) (EctB; EC 2.6.1.76), L-2,4-diaminobutyrate acetyltransferase (EctA; EC 2.3.1.178), and ectoine synthase (EctC; EC 4.2.1.108), with DAB and *N*-γ-acetyl-L-2,4-diaminobutyrate as the respective intermediates (Peters et al., 1990; Ono et al., 1999; Reshetnikov et al., 2011). In a substantial subgroup of ectoine producers (Prabhu et al., 2004; Garcia-Estepa et al., 2006; Bursy et al., 2007; Czech et al., 2018a), ectoine is modified in a position- and stereo-specific reaction by the ectoine hydroxylase (EctD; EC 1.14.11.55) (Inbar et al., 1993; Bursy et al., 2007; Höppner et al., 2014). The resulting 5-hydroxyectoine often possesses superior function-preserving attributes when compared with ectoine or it is preferentially produced during stationary phase (Borges et al., 2002; Manzanera et al., 2004; Schiraldi et al., 2006; Bursy et al., 2007; Harishchandra et al., 2010; Tanne et al., 2014; Abdel-Aziz et al., 2015).

The ectoine biosynthetic genes (*ectABC*) (Louis and Galinski, 1997) are frequently organized in an operon but other genetic arrangements are also known (Schwibbert et al., 2011; Czech et al., 2018a; Leon et al., 2018). Typically, the transcription of *ect* gene clusters is osmotically inducible (Kuhlmann and Bremer, 2002; Czech et al., 2018a, b; Stiller et al., 2018). *ect* gene clusters may contain the gene for the ectoine hydroxylase (*ectD*) and sometimes also for a specialized aspartokinase (*ask_ect*) (Reshetnikov et al., 2006; Lo et al., 2009; Stöveken et al., 2011). Aspartokinase (Ask), and in selected cases also the aforementioned Ask_Ect enzyme, along with L-aspartate-β-semialdehyde-dehydrogenase (Asd), provide the precursor L-ASA for ectoine biosynthesis (Peters et al., 1990; Bestvater et al., 2008; Reshetnikov et al., 2011; Czech et al., 2018a). In addition to ectoine biosynthesis, various types of transport systems mediate the acquisition of these stress protectants from scarce environmental sources with high affinity and in an energy-favorable fashion (Jebbar et al., 1992; Peter et al., 1998; Oren, 1999; Grammann et al., 2002; Vermeulen and Kunte, 2004; Kuhlmann et al., 2011). Transporters also enable uptake of ectoines when these nitrogen-rich compounds (Figure 1) are exploited as nutrients (Jebbar et al., 2005; Schwibbert et al., 2011; Schulz et al., 2017).

Two of the ectoine/5-hydroxyectoine biosynthetic enzymes, namely, the ectoine synthase EctC and the ectoine hydroxylase EctD, have already been studied in detail through biochemical and structural analysis (Bursy et al., 2007; Witt et al., 2011; Höppner et al., 2014; Widderich et al., 2014a, b, 2016a,b; Czech et al., 2019). However, the properties of the two other enzymes of the ectoine biosynthetic pathway, the L-2,4-diaminobutyrate TA EctB and the DAB acetyltransferase EctA, are far less well understood. Here we focus on EctB.

The EctB enzyme catalyzes the first step of ectoine production (Peters et al., 1990; Ono et al., 1999; Reshetnikov et al., 2011; Figure 1). However, only the EctB enzyme from *H. elongata* has been biochemically characterized to some extent (Ono et al., 1999) and rather preliminary data were also reported for the corresponding enzyme from *Methylomicrobium alcaliphilum* (Reshetnikov et al., 2006, 2011). EctB enzymes belong to the superfamily of TAs (also referred to in the literature as aminotransferases) and depend on pyridoxal-5′-phosphate (PLP) as their cofactor (Steffen-Munsberg et al., 2015). During ectoine biosynthesis, EctB uses L-glutamate as the amino donor and L-ASA as the acceptor molecule to form 2-oxoglutarate and DAB. DAB then serves as the substrate of the DAB acetyltransferase EctA, which catalyzes the second step of the ectoine biosynthetic route (Peters et al., 1990; Ono et al., 1999; Czech et al., 2018a; Figure 1).

To extend our knowledge of EctB as a critical player of ectoine biosynthesis, we have biochemically characterized the EctB protein from the thermo-tolerant Gram-positive bacterium *Paenibacillus lautus* (*Pl*). Our biochemical analysis shows that this homo-tetrameric protein is highly tolerant to temperature, pH, and salt and that its catalytic activity is strictly dependent on PLP. We provide insights into the EctB reaction mechanism based on structural modeling and biochemical validation and show that EctB enzymes act in a way analogous to gamma-aminobutyrate TAs (GABA-TAs). Our phylogenomic analysis of the *ect* genes in many members of the Paenibacilli, including *P. lautus*, revealed an unusual genetic juxtaposition of the ectoine biosynthetic genes with those for an Ehu-type ectoine/5-hydroxyectoine binding-protein-dependent ABC transport system. These findings indicate that these microorganisms genetically coordinated the biosynthesis of ectoines with the scavenging of these versatile stress protectants from environmental sources.

## Materials and Methods

### Chemicals

L-aspartate-β-semialdehyde was purchased from GlycoSyn (Lower Hutt, New Zealand). Desthiobiotin, Strep-TactinSuperflow chromatography material for the purification of proteins fused to a *Strep*-tag II affinity peptide and anhydrotetracycline hydrochloride (AHT) for the induction of enhanced transcription of the TetR-regulated *tet* promoter present on the parent plasmid pASG-IBA5 and its (*Pl*)*ectB* carrying derivatives, plasmids pAR8 and pAR16, were purchased from IBA GmbH (Göttingen, Germany). Other chemicals, including PLP and DAB, were obtained from Sigma–Aldrich (Taufkirchen, Germany) and Roth (Karlsruhe, Germany). The protein stain InstantBlue was purchased from Expedeon (Heidelberg, Germany).

### Recombinant DNA Procedures and Construction of Plasmids

The DNA sequence of the *ectB* gene was retrieved from the genome sequence (accession number: NC_013406.1) of the *P. lautus* strain Y412MC10 (Mead et al., 2012) and was used as the template for the synthesis of a codon-optimized version of (*Pl*)*ectB* for its recombinant expression in *Escherichia coli*. This synthetic (*Pl*)*ectB* gene construct was purchased from Invitrogen GeneArt (Thermo Fisher Scientific, Waltham, MA, United States); its DNA sequence was deposited in the NCBI database under accession number MK682511.1 (*ectB*)^1^. To allow the overproduction and affinity purification of the recombinant *P. lautus* EctB protein in *E. coli*, we constructed a hybrid (*Pl*)*ectB* gene that encodes a (*Pl*)EctB protein carrying at its amino-terminus a *Strep*-tag II affinity peptide [NH_2_-W-S-H-P-Q-F-E-K-(S-G)]. For this purpose, the codon-optimized (*Pl*)*ectB* gene was amplified by PCR from the plasmid (pLC47) provided by the supplier of the synthetic construct using custom-synthesized primers (pLC47_for and pLC47-rev; Supplementary Table S1). The resulting DNA fragment was used for the construction of the (*Pl*)*ectB* expression plasmid pLC52 by applying Stargate combinatorial cloning technology (IBA GmbH, Göttingen, Germany). Hence, the (*Pl*)*ectB* gene was first introduced into a pENTRY vector^2^ resulting in plasmid pLC49. By further relying on Stargate combinatorial cloning technology (IBA GmbH, Göttingen, Germany), the (*Pl*)*ectB* gene was subsequently inserted into the expression plasmid pASG-IBA5^3^, which allows (*Pl*)*ectB* gene expression under the control of the *tet* promoter carried by the plasmid; this plasmid was called pLC52. The transcriptional activity of the *tet* promoter is controlled by the AHT-responsive TetR repressor, whose structural gene is carried by the plasmid-backbone of pASG-IBA5. A cleavage site for the Xa-protease was introduced into the codon optimized (*Pl*)*ectB* gene present on plasmid pLC52 by site-directed mutagenesis using the Q5 Site-Directed Mutagenesis Kit (New England BioLabs GmbH, Frankfurt a. M., Germany) with custom synthesized DNA primers (pair Strep_XA_EctB_Mut_R and Strep_XA_EctB_Mut_F2; Supplementary Table S1). The resulting plasmid was pAR8; a map of this plasmid is shown in Supplementary Figure S1. The linker sequence connecting the *Strep*-tag II affinity peptide [NH_2_-W-S-H-P-Q-F-E-K] to the N-terminus of (*Pl*)*EctB* was S-G-M-I-E-G-R. In addition, the following (*Pl*)*ectB* variants were constructed via site-directed mutagenesis of pAR8: plasmids pAR16 (AAA/CAT; Lys^274^/His), pAR18 (AAA/GCG; Lys^274^/Ala), and pAR19 (AAA/CGT; Lys^274^/Arg) (for the sequence of the DNA-primers used for the site-directed mutagenesis experiments, see Supplementary Table S1). The correct nucleotide sequence of the wild-type (*Pl*)*ectB* gene in all constructed plasmids was ascertained by DNA sequence analysis [Eurofins MWG Operon (Ebersberg, Germany)] and all primers used for plasmid or (*Pl*)*ectB* mutant constructions were purchased from Microsynth AG (Lindau, Germany) (Supplementary Table S1).

### Bacterial Strains, Media, and Growth Conditions

For the routine propagation of plasmids carrying the (*Pl*)*ectB* gene, we used the *E. coli* strain TOP10 (Invitrogen, Carlsbad, CA, United States). Cells were grown at 37°C in Luria–Bertani (LB) liquid medium containing ampicillin (100 μg ml^–1^) to select for the presence of plasmids. The (*Pl*)EctB protein and its mutant derivatives were heterologously produced in the *E. coli* B strain BL21 (Dubendorff and Studier, 1991) using modified minimal medium A (MMA) (Miller, 1972) containing 0.2% (w/v) glucose as the carbon source and 0.1% (w/v) casamino acids, 1 mM MgSO_4_, and 3 mM thiamine as supplements.

### Overproduction, Purification, and Determination of the Quaternary Assembly of EctB Proteins

Cells of the *E. coli* B strain BL21 harboring the (*Pl*)*ectB-*expression plasmid pAR8, or its mutant derivatives, were inoculated into modified MMA containing 100 μg ml^–1^ ampicillin (1 L medium in a 2-L Erlenmeyer flask) to an OD_578_ of 0.1 from an overnight pre-culture prepared in LB medium. The cultures were grown on an aerial shaker (set to 180 rpm) at 37°C until they reached an OD_578_ of 0.5. Expression of the plasmid-encoded (*Pl*)*ectB* gene was then triggered by the addition of the synthetic TetR inducer AHT to a final concentration of 0.2 mg ml^–1^ resulting in increased (*Pl*)*ectB* expression. After 2 h of further growth, the cells were harvested by centrifugation (2360 × *g* at 4°C for 10 min), re-suspended in 15 mL of purification buffer [100 mM Tris-HCl (pH 7.5), 150 mM NaCl], and then disrupted by passing them three times through a French Pressure cell (at 16,000 psi). A cleared cell lysate of the disrupted cells was prepared by centrifugation (31,870 × *g* at 4°C for 36 min) and the supernatant was filtrated through a Filtropur S 0.45 (Sarstedt, Nümbrecht, Germany) device to remove remaining cellular debris. This cleared cell extract was then used for the purification of the recombinant *Strep*-tag II-marked (*Pl*)EctB protein by affinity chromatography on Strep-Tactin affinity resin as detailed in the protocol of the supplier (IBA GmbH, Göttingen, Germany). A Nanodrop Photospectrometer ND1000 (Peqlab, Erlangen, Germany) was used for determination of the (*Pl*)EctB protein concentration in the elution fractions from the Strep-Tactin Superflow affinity column; for these measurements (at a wavelength of 280 nm), we used an extinction coefficient of 46,410 M^–1^ cm^–1^. The evaluation of the purity of the purified (*Pl*)EctB protein was performed by SDS–PAGE (15% polyacrylamide) with the PageRuler Pre-stained Protein Ladder (Thermo Fisher Scientific) as a reference.

### Analytical Size-Exclusion Chromatography With Multi-Angle Light Scattering

Analytical size-exclusion chromatography (SEC) with multi-angle light scattering (SEC-MALS) was performed at 4°C. A 100 μL sample (at a protein concentration of 100 μM) of purified (*Pl*)EctB (or its mutant Lys^274^/His variant) was injected into an S200 300/10 GL analytical size-exclusion column (GE Healthcare, München, Germany) attached to a multi-angle light scattering (MALS) and a differential refractive index (RI) detector (Postnova Analytics, Landsberg am Lech, Germany). The chromatography column was pre-equilibrated at 4°C with buffer of pH 7.5 containing 20 mM HEPES, 200 mM NaCl, 20 mM MgCl_2_, and 20 mM KCl.

### EctB Enzyme Activity Assays

Due to the limited commercial availability of the substrate L-ASA to measure the kinetic parameters of the forward EctB enzyme reaction (Ono et al., 1999), the reverse reaction of this enzyme was assayed as described previously using the production of L-glutamate as readout for the detection of enzyme activity and its quantification via HPLC analysis (Reshetnikov et al., 2006, 2011). The loading of (*Pl*)EctB with the co-factor PLP was tested in 100 μL 100 mM Tris-HCl buffer (pH 7.0) containing 100 mM NaCl, 10 mM DAB, 15 mM 2-oxoglutarate, 2.7 μg purified enzyme, and varying concentrations of PLP. All reaction components, except for the enzyme and the cofactor PLP, were pre-incubated at the reaction temperature of 45°C. After 1 min, the (*Pl*)EctB-mediated reaction was stopped by adding 100 μL acetonitrile to the reaction solution. The determination of the basic parameters of (*Pl*)EctB was performed with the same assay in the presence of 1 mM PLP and 5 μg purified enzyme at 45°C; the runtime of the assay was 1 min. For the temperature screening (10–60°C), the Tris buffer was adjusted to pH7.0 with HCl at the particular temperature at which the (*Pl*)EctB enzyme activity was tested. The screening buffer for the pH-optimum of the (*Pl*)EctB enzyme consisted of a buffer mixture [MES-HCl (pH 5.5), Tris-HCl (pH 7.5), CHES-NaOH (pH 10)] with 50 mM each, adjusted with 37% HCl or 5 M NaOH at 45°C. The pH buffer range of MES is 5.5–6.7, that of Tris is 7.0–9.0, and that of CHES is 8.6–10.0. The screening assay for the salt dependency of (*Pl*)EctB enzyme activity contained varying concentrations (0–1.5 M) of NaCl or KCl, respectively. The reactions were stopped by the addition of 100 μL acetonitrile after 2 min of reaction time.

The kinetic parameters of the (*Pl*)EctB enzyme were determined using the assay described above with either 10 mM DAB and varied concentrations of 2-oxoglutarate (0.1–30 mM), or with 15 mM 2-oxoglutarate and varied concentrations of DAB (0.1–15 mM). The reaction time chosen for these enzyme assays was 1 min, conditions under which the enzyme activity proceeded linearly (Supplementary Figure S2). The enzyme activity of the various (*Pl*)EctB mutants with a substituted Lys^274^ residue (either His, Ala, or Arg) was assayed in a 100 μL reaction containing 100 mM Tris-HCl buffer (pH 7.0), 100 mM NaCl, 10 mM DAB, 15 mM 2-oxoglutarate, 1 mM PLP, and 5 μg purified enzyme at 45°C. The enzyme reactions of the wild-type (*Pl*)EctB protein and its three variants were stopped after 5 min by the addition of 100 μL acetonitrile. This longer reaction time was chosen to detect even marginal enzyme activities of the (*Pl*)EctB variants. The enzyme activities of the (*Pl*)EctB mutants were benchmarked against the wild-type enzyme whose activity was set to 100%. None of the three tested (*Pl*)EctB variants exhibited any enzyme activity. All (*Pl*)EctB assays were performed using two independently produced and purified protein preparations, and each protein sample was assayed twice.

### HPLC Analysis of the “Reverse Reaction” Product Glutamate

The product, L-glutamate, of the (*Pl*)EctB reverse enzyme reaction (Reshetnikov et al., 2011) was derivatized with ortho-phthalaldehyde (OPA) using a procedure based on previously published methods (Reshetnikov et al., 2011). In brief: 3 μL of the reaction sample was added to 6 μL borate buffer (0.4 M, pH 10.2) and subsequently mixed with 1 μL of an OPA solution [borate buffer (0.4 M, pH 10.2) containing 50% MeOH, 0.65% 3-mercaptopropionic acid, and 10 mg mL^–1^ OPA] in a thermomixer (1 min, 900 rpm, at 20°C). The solution was then diluted in 65 μL H_2_O, centrifuged (20,800 × *g* for 10 min at room temperature) to remove the denatured (*Pl*)EctB protein; 50 μL of the supernatant was injected into an HPLC system (1260 Infinity; Agilent Technologies, Walsbronn, Germany) using a 150 × 4.6 mm Gemini^®^ 5μM C18 110 Å column (Phenomenex, Aschaffenburg, Germany) and an attached fluorescence detector (Agilent Technologies, Walsbronn, Germany). The fluorescence detector was set to an excitation wavelength of 340 nm and an emission wavelength of 450 nm. For the mobile phase for the HPLC column, the following solvents were used—A: phosphate buffer [40 mM, pH 7.8] and B: 45% methanol and 45% acetonitrile in water. The separation of the OPA derivatives was achieved using a flow rate of 1 mL min^–1^ at 40°C and the following gradient of solvents A and B [at min 0–100% of solvent A and 0% of solvent B; at min 10–90% of solvent A and 10% of solvent B; at min 12–0% of solvent A and 100% of solvent B].

### HPLC-MS Analysis of L-ASA

High-pressure liquid chromatography mass spectrometry (HPLC-MS)/MS analysis of L-ASA was performed on an Agilent 6495B Triple Quad LC/MS system equipped with an electrospray ionization source. The solvent system used was water (A) and acetonitrile (B), both supplemented with formic acid to a final concentration of 0.1%. Samples were kept at 15°C and the temperature of the column oven was maintained at 40°C. The general MS source parameters were as follows: Capillary voltage was set at 3 kV and nitrogen gas was used as nebulizing (25 psig), drying (11 L min^–1^ 130°C) and sheath gas (12 L min^–1^, 400°C). LC-MS data were analyzed and quantified using MassHunter Qualitative Navigator and QQQ Quantitative Analysis software (Agilent). L-ASA was derivatized according to the method described by Han et al. (2013). Briefly, 50 μL of sample was mixed with 50 μL 150 mM 1-(3-dimethylaminopropyl)-3-ethylcarbodiimide, 50 μL 250 mM 3-nitrophenylhydrazine (3-NPH), and 50 μL of 7.5% pyridine in methanol in a 1.5 mL Eppendorf tube. The reaction was incubated at 30°C for 30 min. After incubation, the samples were centrifuged at 13,000 × g for 1 min and the supernatant was transferred into HPLC vials. The derivatized analytes were separated on an RP 18 column (50 mm × 2.1 mm, particle size 1.7 μm, Kinetex EVO C18, Phenomenex, Aschaffenburg, Germany). The gradient was as follows: 0 min 5% B; 1 min 5% B, 6 min 95% B; 6.5 min 95% B; 7 min 5% B at a flow rate of 250 μL/min. Samples were held at 15°C and the injection volume was 5 μL. MRM data were acquired in negative mode. Optimized collision energies used for the 3-NPH derivatized L-ASA were 386 *m/z* 207 *m/z* (25 V) and 386 *m/z* 137 *m/z* (35 V). The dwell time and fragmentor voltage were 100 ms and 380 V, respectively.

### Assessing the Substrate Specificity of the (*Pl*)EctB Protein

Since the EctB-catalyzed reverse reaction is similar to the forward reaction catalyzed by GABA-TAs (Bruce et al., 2012; Steffen-Munsberg et al., 2015), we assayed (*Pl*)EctB enzyme activity for use of the substrate GABA. For this purpose, we used the following enzyme reaction mixture: 100 mM Tris-HCl (pH 7.0), 100 mM NaCl, 15 mM 2-oxoglutarat, 2.7 μg purified enzyme, and 10 mM DAB or GABA (10 or 2 mM, respectively). The reaction was performed at 45°C and stopped after 1 min reaction time by adding acetonitrile. Synthesis of L-glutamate with either DAB or GABA as the substrate was used as read-outs for the (*Pl)*EctB catalyzed enzyme reactions. The formed L-glutamate was quantified by HPLC analysis after modification by OPA as described above.

### Bioinformatics Analysis of EctB- and EhuB-Type Proteins

The *in silico* analysis of EctB-type enzymes was based on a recent analysis of the phylogenomic distribution of *ect* biosynthetic gene clusters in *Bacteria* and *Archaea*. This manually curated dataset of ectoine biosynthetic genes contained 437 microbial species/strains (Czech et al., 2018a); seven *ectB* genes were removed from this original dataset because the *ectB* genes of these microorganisms contained large deletions. EhuB-type substrate-binding proteins of ABC transporters for ectoine/hydroxyectoine (Jebbar et al., 2005; Hanekop et al., 2007) were identified by a blast search of all available *Paenibacillus* genomes at the Joint Genome Institute (JGI) Integrated Microbial Genomes and Microbiomes (IMG/M)^4^ database (Chen et al., 2019), using the (*Pl*)EctC protein sequence as the query to identify *ect* biosynthetic gene clusters. Subsequently, the gene neighborhoods of the *ect* gene clusters were analyzed with tools provided by the IMG/M-webserver by scoring the presence of nearby *ehuABCD*-type genes (Jebbar et al., 2005; Hanekop et al., 2007). The comparison of amino acid sequences of proteins related to the *Sinorhizobium meliloti* EhuB substrate-binding protein (Jebbar et al., 2005; Hanekop et al., 2007) was performed with the MAFFT multiple amino acid sequence alignment tool^5^ (Katoh et al., 2017).

### Generation of Model Structures of (*Pl*)EctB and (*Pl*)EhuB Proteins and Preparation of Figures

Using the amino acid sequences of the (*Pl*)EctB and (*Pl)*EhuB proteins, *in silico* models of these proteins were generated via the SWISS-MODEL server^6^ (Waterhouse et al., 2018). For the (*Pl*)EctB protein, the crystal structures of the *Arthrobacter aurescerns* GABA-TA (PDB-ID: 4ATQ and 4ATP) (Bruce et al., 2012) were used as the template. For the (*Pl*)EhuB protein, the SWISS-MODEL server automatically chose the crystal structure of the *S. meliloti* EhuB protein in complex with ectoine (PDB-number: 2Q88) (Hanekop et al., 2007) as the template. Figures of the crystal structure of the (*Aa*)GABA-TA protein, and of models of the (*Pl*)EctB and (*Pl*)EhuB proteins were prepared using Chimera^7^ (Pettersen et al., 2004) or PyMol^8^ (Delano, 2002).

### Molecular Docking of Ectoine Into the *in silico* Model of (*Pl*)EhuB Substrate Binding Protein

Ectoine was docked into the active site of the (*Pl*)EhuB model that had been generated with the SWISS-MODEL server (Waterhouse et al., 2018) with the program AUTODOCK using standard settings (Trott and Olson, 2010). Two different orientations of the ectoine molecule were initially given as input to circumvent any bias; these two orientations represented a 180° flip of the ectoine molecule. The output of the docking process was manually inspected and clashing of the ectoine molecule with side chains of the (*Pl*)EhuB model were manually checked using the program COOT (Emsley and Cowtan, 2004).

## Results

### EctB Is a Homotetramer and Binds PLP

**FIGURE 2:**
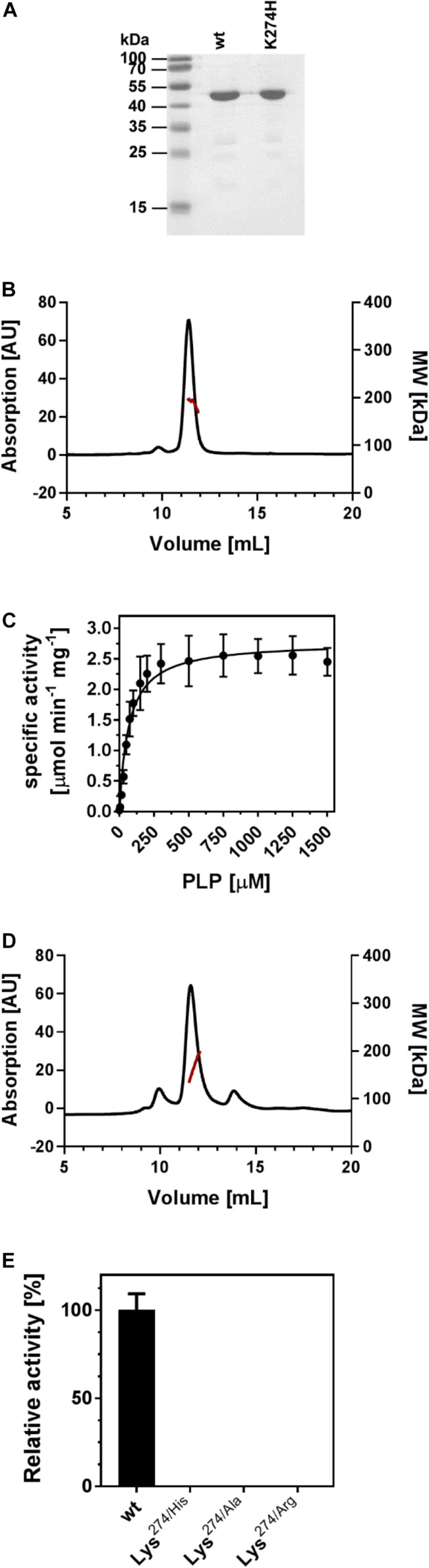
EctB is a homotetramer and its enzyme activity is strictly dependent on PLP. **(A)** Commassie-stained SDS-PAGE of the purified (*Pl*)EctB wild-type protein and its Lys^274/His^ variant. The size standard is given in kilo dalton (kDa). **(B)** SEC chromatogram of an EctB purification shows that the protein elutes in a single peak. MALS-RI analysis (red line) shows that the EctB protein (49.1 kDa) elutes with an absolute molecular mass of approximately 200 kDa, consistent with the notion that the (*Pl*)EctB protein is a homotetramer in solution. **(C)** The specific activity of (*Pl*)EctB depends on the presence of PLP. The error bars represent the standard deviation and were calculated from two technical and two biological replicates. **(D)** SEC chromatogram of an EctB Lys^274/His^ variant. MALS-RI analysis (red line) shows that EctB Lys^274/His^ variant elutes with an absolute molecular mass consistent with that of forming a homotetramer in solution. **(E)** The activity of (*Pl*)EctB depends on Lys^274^. The relative activities of (*Pl*)EctB and three different (*Pl*)EctB Lys^274^ variants show that this amino acid residue is essential for enzyme activity. The enzyme activity of the (*Pl*)EctB wild-type protein was set to 100%. Under the tested conditions, (*Pl*)EctB showed an activity of 2.45 ± 0.16 U mg^–1^protein; one unit (U) is defined as the enzymatic conversion of 1 μmol 2-oxoglutarate to 1 μmol glutamate min^–1^. Error bars represent the standard deviation of two technical and two biological replicates.

The *P. lautus* strain Y4.12MC10 has been isolated from the effluent of a hot spring in the Yellowstone National Park (United States) and can grow at temperatures of up to 50°C under laboratory conditions (Mead et al., 2012). It possesses a canonical *ectABCD* gene cluster (Czech et al., 2018a). We used the *ectB* gene from this thermotolerant Gram-positive bacterium as the template for the synthesis of a codon-optimized *ectB* version (accession number MK682511.1) to enhance the synthesis of (*Pl*)EctB in *E. coli*. We constructed an expression vector containing the recombinant (*Pl*)*ectB* gene so that the produced (*Pl*)EctB enzyme would contain a N-terminal *Strep*-tag II peptide for affinity purification. The recombinant (*Pl*)EctB protein was overproduced in *E. coli* BL21 and was subsequently purified by streptavidin-affinity chromatography (Figure 2A). The purified (*Pl*)EctB protein has a calculated molecular mass of 49.1 kDa, including the *Strep*-tag II peptide and its connecting linker sequence. MALS coupled to RI experiments unambiguously showed that the protein elutes from the SEC column with an absolute molecular mass of approximately 200 kDa, suggesting that (*Pl*)EctB forms a homotetramer in solution (Figure 2B).

### The PLP-Dependent (*Pl*)EctB Protein Is a Highly Robust Enzyme

Next, we characterized the enzymatic properties of (*Pl*)EctB. Enzyme activity of EctB is known to be dependent on the co-factor PLP (Ono et al., 1999; Reshetnikov et al., 2011). However, contrary to expectations, the recombinant (*Pl*)EctB protein did not possess the characteristic yellow color of PLP-containing enzymes (Steffen-Munsberg et al., 2015; Richts et al., 2019), thereby preventing its photospectrometric analysis. To study the predicted PLP-dependency of (*Pl*)EctB, we first incubated (*Pl*)EctB with increasing amounts of PLP and analyzed its ability to convert DAB into L-glutamate (Figure 2C). As reported previously (Ono et al., 1999; Reshetnikov et al., 2011; Chen et al., 2015), we decided to measure the “reverse reaction” of the EctB enzyme (Figure 1), because the substrate of the physiologically relevant forward reaction, L-ASA, has so far not been commercially available in concentrations suitable for a complete set of kinetic analysis. We used the formation of L-glutamate as the readout of the reverse reaction catalyzed by the (*Pl*)EctB enzyme. Our experiments demonstrated that the specific activity of (*Pl*)EctB strictly relies on the addition of PLP (Figure 2C). Apparently, the (*Pl*)EctB protein is produced in the heterologous *E. coli* host as an apo-protein. Approximately 300 μM of PLP was required in order to achieve maximum (*Pl*)EctB activity. Since our experiments show that enzyme activity of the recombinant (*Pl*)EctB protein can be recovered by PLP addition (Figure 2C), we added PLP to all following enzyme assays.

**FIGURE 3:**
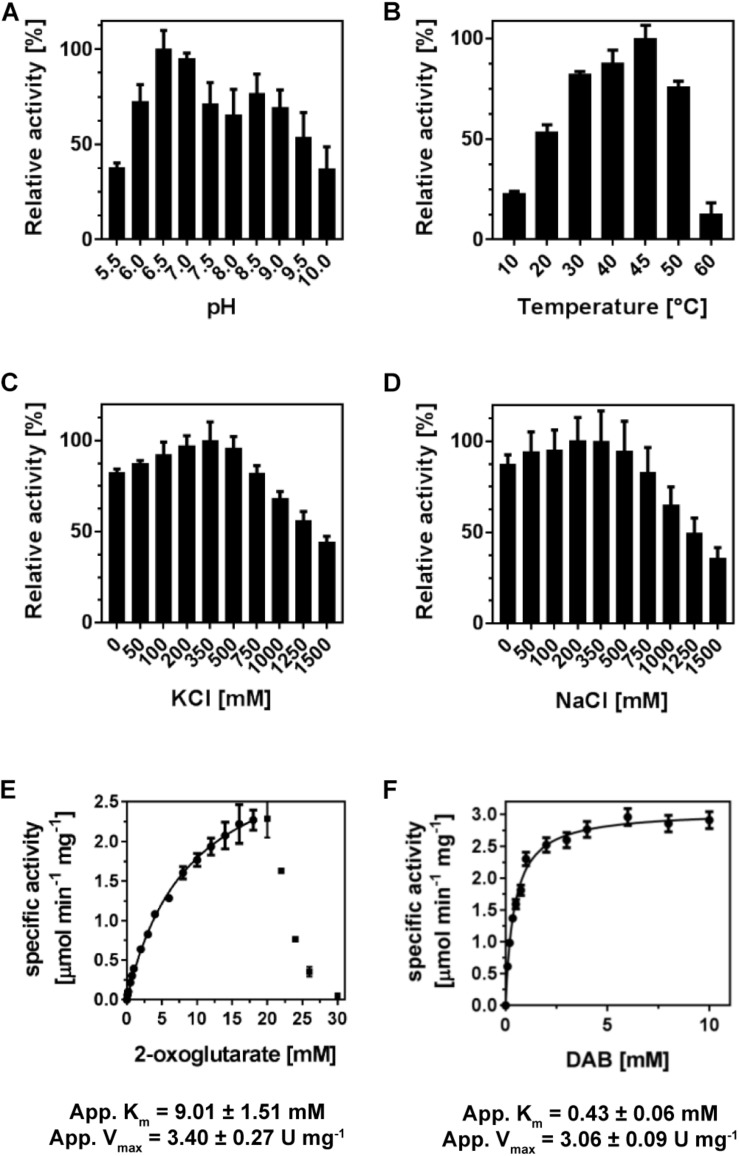
Enzyme-kinetics of (*Pl*)EctB. The enzyme activity of (*Pl*)EctB was determined with respect to its **(A)** pH optimum, **(B)** temperature, and its salt tolerance against KCl **(C)** and NaCl **(D)**. The velocity (v) of (*Pl*)EctB at increasing concentrations of the 2-oxogultarate **(E)** and DAB **(F)** shows that the enzyme efficiently catalyzes the “reverse reaction.” For **A**–**F**: Error bars represent the standard deviation calculated from two technical and two biological replicates.

We analyzed the specific activities of (*Pl*)EctB with respect to pH, temperature, and sodium and potassium chloride (Figure 1). (*Pl*)EctB has a broad tolerance to pH variations and operates best in a temperature range of 30–50°C (Figures 3A,B). (*Pl*)EctB showed similar activities in the presence of increasing concentrations of sodium and potassium chloride (Figures 3C,D). Taken together, (*Pl*)EctB is a highly robust enzyme with respect to variations in pH, temperature, and salt concentration. We note in this context that the purified *H. elongata* EctB protein required substantial concentrations of K^+^ for its stability and activity (Ono et al., 1999). The (*Pl*)EctB protein, in contrast, was active in the absence of KCl and there was also no strong difference whether KCl or NaCl was present in the assay solutions (Figures 3C,D).

### Kinetic Analysis of the Reverse Reactions of (*Pl*)EctB

Based on these initial data, we devised an optimal enzyme assay containing 100 mM Tris-HCl buffer (pH 7.0), 100 mM NaCl, 10 mM DAB, 15 mM 2-oxoglutarate, and 1 mM PLP; we assayed the enzyme activity at a temperature of 45°C and for 1 min as enzyme activity of (*Pl*)EctB progresses linearly in this time span (Supplementary Figure S2). (*Pl*)EctB exhibited an apparent *K*_m_ for 2-oxoglutarate of 9 ± 1.5 mM and a *V*_max_ of 3.4 ± 0.3 U mg^–1^, as monitored by the production of L-glutamate (Figure 3E). We note, that at concentrations exceeding 20 mM 2-oxoglutarate in the enzyme assay, the activity of (*Pl*)EctB decreases drastically (Figure 3E). This could possibly be caused by the high concentration of 2-oxoglutarate in the assay buffer. Alternatively, during the EctB-mediated catalysis of the reverse reaction, the substrates for the forward reaction (L-glutamate and L-ASA) will inevitably be formed (Figure 1). Consequently, at high substrate concentrations for the reverse reaction (Figure 3E), the expected accumulation of L-glutamate and L-ASA (the substrates for the forward reaction) could potentially interfere with the measured activity of the reverse reaction. It is known from the kinetic characterization of the *H. elongata* EctB enzyme (Ono et al., 1999) that the velocity of the forward reaction is approximately fourfold higher then that of the backward reaction that we measured here for the (*Pl*)EctB enzyme (see Table 1 for comparative data).

**TABLE 1:** Enzyme-kinetic properties of the EctB enzymes from *H. elongata* and *P. lautus*.

**Substrate**	***K*_m_ (mM)**	***V*_max_ (μmol min^–1^ mg^–1^)**	**References**
(*He*)EctB–L-glutamate^a^	9.1	12	Ono et al., 1999
(*He*)EctB–D,L-ASA^a^	4.5	12	Ono et al., 1999
(*Pl*)EctB–2-oxoglututate^b^	9.0	3.4	This study
(*Pl*)EctB–DAB^b^	0.4	3.1	This study

*^*a*^Substrate of (*He*)EctB for the forward reaction. ^*b*^substrate of (*Pl*)EctB for the “reverse” reaction.*

With DAB as the varied substrate in the enzyme assay, (*Pl*)EctB showed a Michaelis-Menten behavior with an apparent *K*_m_ of 0.40 ± 0.01 mM and a *V*_max_ of 3.10 ± 0.01 U mg^–1^ (Figure 3F). To verify that the “reverse reaction” of (*Pl*)EctB indeed produced L-ASA besides L-glutamate (Figure 1), we monitored its appearance by HPLC-MS. L-ASA was indeed produced by (*Pl*)EctB in the “reverse reaction” (Supplementary Figure S3). Collectively, these experiments showed that both L-ASA and L-glutamate are produced in the “reverse reaction” (Figure 3 and Supplementary Figure S3), re-confirming that the used assay is a valid approach to analyze the activity of the (*Pl*)EctB enzyme (Ono et al., 1999; Reshetnikov et al., 2011; Chen et al., 2015).

Compared to data reported by Ono et al. (1999) for the forward reaction of the *H. elongata* enzyme, the apparent *K*_m_ values of the (*Pl*)EctB enzyme in its “reverse reaction” are similar, but the *V*_max_ values of these two enzymes differ by about fourfold (Table 1).

### Lys^274^ Attaches the PLP Cofactor to (*Pl*)EctB

The unabated increase in the number of crystallographic structures in the Protein Database (PDB) (Burley et al., 2017), allowed the development of modeling servers such a SWISS-Model^9^ (Waterhouse et al., 2018) and Phyre2^10^ (Kelley et al., 2015) to aid the prediction of protein structures from the amino acid sequence. This is a widely used approach in molecular biology and biochemistry to dissect *in silico* the salient features of proteins of interest and probe these models by biochemical and biophysical techniques (Kelley et al., 2015; Waterhouse et al., 2018).

When we queried the SWISS-MODEL server (Waterhouse et al., 2018) with the (*Pl*)EctB amino aid sequence, the system automatically proposed the GABA-TA from *E. coli* and *Arthrobacter aurescens* (Liu et al., 2004, 2005; Bruce et al., 2012) as the most suitable templates for the construction of an *in silico* model of the (*Pl*)EctB protein. We chose the crystal structure of the *A. aurescens* GABA-TA as the template for this this purpose because it contained the PLP-GABA adduct, a crucial intermediate in the GABA-TA-mediated enzyme reaction (Bruce et al., 2012; Steffen-Munsberg et al., 2015).

**FIGURE 4:**
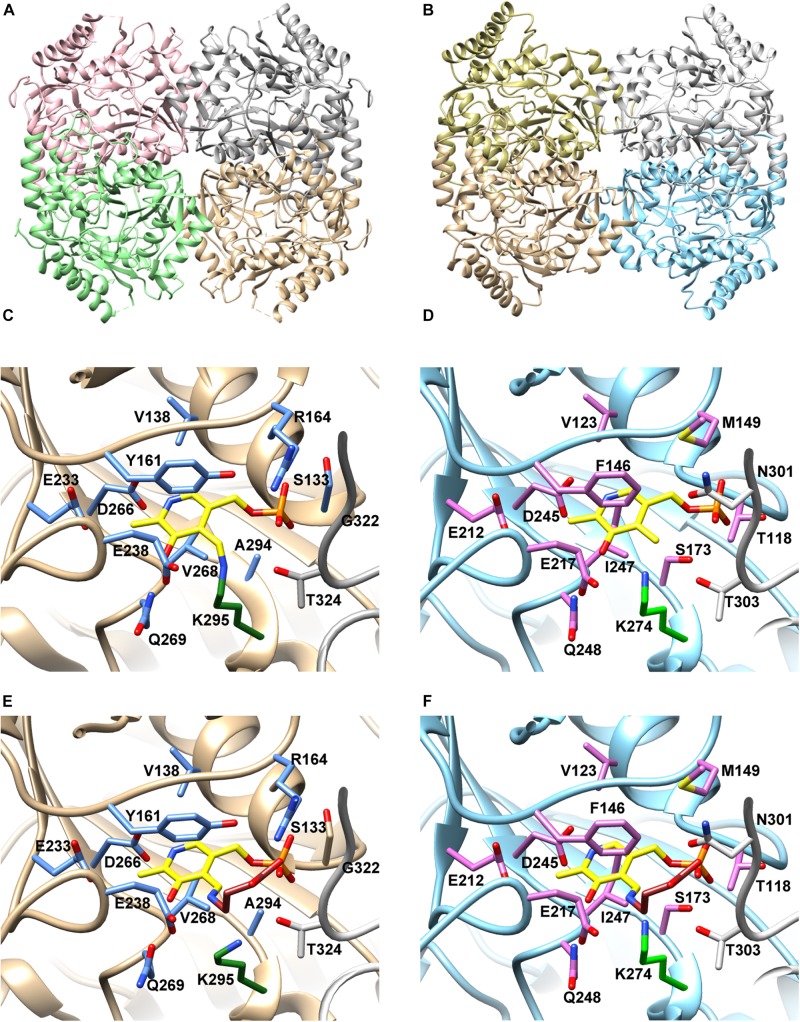
Structural model of (*Pl*)EctB. **(A)** Crystal structure of the GABA-TA from *A. aurescens* (PDB-ID: 4ATP) and **(B)** the SWISS-MODEL of (*Pl*)EctB. Different color schemes highlight monomeric subunits within the tetrameric assembly of *A. aurescens* GABA-Ta **(A)** and in the model of the (*Pl*)EctB aminotransferase **(B)**. **(C)** The architecture of the PLP-binding site, formed by two monomeric subunits, of (*Aa*)GABA-TA (PDB-ID: 4ATP) shows the PLP-binding amino acid residues including the covalent binding of PLP (yellow) to Lys^295^ (green) forming the internal aldemine (Bruce et al., 2012). The side-chains of amino acid residues implicated in the binding of PLP **(C)** and of the internal aldemine (the PLP-GABA adduct) are shown in blue and gray in stick format. **(D)** Overlay of (*Aa*)GABA-TA in its internal aldemine form with the (*Pl*)EctB model illustrates the similarity of the active sites from both enzymes, identifying Lys^274^ in (*Pl*)EctB as the residue to which PLP (yellow) is bound. The side-chains of amino acid residues implicated in the binding of PLP **(C)** and of the internal aldemine are shown in magenta and gray in stick format. **(E)** Crystal structure of (*Aa*)GABA-TA in the PLP-GABA-adduct bound form (PDB-ID: 4ATQ) (the external aldemine). Note: the carboxyl-group of GABA is coordinated via interactions with the side chain of Arg^164^. **(F)** An overlay of (*Aa*)GABA-TA in its external aldemine form with the (*Pl*)EctB model. Arg^164^ of (*Aa*)GABA-TA is replaced in (*Pl*)EctB by a conserved Met^149^ residue from the same subunit. A loop from the adjacent sub-unit (depicted in gray) contributes to the formation of the binding site in (*Aa*)GABA-TA and involves residues Gly^322^ and Thr^324^ (Bruce et al., 2012). In the (*Pl*)EctB model, residues Asn^301^ and Thr^303^ might serve this function.

The *A. aurescens* GABA-TA enzyme is a tetramer (Figure 4A). In its crystal structure, each monomer contains a PLP cofactor covalently attached to Lys^295^ (PDB-ID: 4ATP) (Figure 4C), thereby forming the internal aldemine characteristic for PLP-dependent TAs (Bruce et al., 2012). Figure 4E shows the active site of the *A. aurescens* GABA-TA enzyme with the PLP-GABA adduct (PDB-ID: 4ATQ), the external aldemine (Bruce et al., 2012). In Figures 4B,D,F, we show the corresponding models of the (*Pl*)EctB protein generated with the SWISS-MODEL server (Waterhouse et al., 2018). The so-derived model of a (*Pl*)EctB monomer superimposes very well with the X-ray structure of the *A. aurescens* GABA-TA protein (Bruce et al., 2012) with a root-mean-square deviation (RMSD) of 0.854 Å over 1852 atoms (Supplementary Figures S4A,B). This was also true for all residues involved the coordination of the PLP cofactor (Supplementary Figure S4C). Building on this model and on an amino acid sequence alignment of the (*Pl*)EctB protein with the related GABA-TA proteins from *A. aurescens* and *E. coli* (Liu et al., 2004, 2005; Bruce et al., 2012; Supplementary Figure S5), we identified Lys^274^ as the most-likely residue for the attachment of the PLP cofactor to (*Pl*)EctB.

To challenge this prediction, we substituted Lys^274^ with His, Ala, or Arg residues, all of which should be unable to attach PLP to (*Pl*)EctB. All the three EctB-Lys^274^ variants were enzymatically inactive (Figure 2E), thus experimentally buttressing the prediction that Lys^274^ attaches the PLP cofactor to the EctB protein. As an example, we studied the EctB-Lys^274/His^ variant further to ascertain that this amino acid substitution did not affect the quaternary assembly of the mutant protein. Using MALS-RI experiments. We found that EctB-Lys^274/His^, like the parent (*Pl*)EctB protein, forms a tetramer in solution (Figure 2D). Taken together, our analysis shows that (*Pl*)EctB forms a homotetramer with Lys^274^ attaching, in all likelihood, the catalytically important cofactor PLP to each of the monomers.

### EctB-Type Proteins Are Highly Conserved

With the biochemical characterization of the first EctB protein from a thermotolerant Gram-positive bacterium in hand (Figures 2, 3), we now wished to compare the conservation of EctB proteins among phylogenetically diverse microorganisms. Building on a previously reported curated dataset of *bona-fide* ectoine biosynthetic gene clusters (Czech et al., 2018a), we found that the degree of amino acid sequence identity of the compared 430 EctB proteins ranged between 91% (for *Paenibacillus glucanolyticus* DSM 5162) and 51% (for *Kytococcus sedentarius* DSM 20547) when we used the (*Pl*)EctB protein as search query. We found that 52 amino acid residues among the aligned 430 EctB proteins are strictly conserved. As expected from our data on PLP-binding by (*Pl*)EctB (Figures 2C,E), Lys^274^ is among them. An abbreviated amino acid sequence alignment of 10 randomly chosen bacterial and archaeal EctB protein from the above-described collection is shown in Supplementary Figure S6.

While ectoine/5-hydroxyectoine biosynthetic genes are widely found in *Bacteria*, they are rarely present in archaeal genomes (Widderich et al., 2016a; Qin et al., 2017; Czech et al., 2018a). Current evidence suggests that these latter microorganisms have acquired them via lateral gene transfer events (Widderich et al., 2016a; Ren et al., 2019). The uneven distribution of *ect* gene clusters in taxonomically closely related members of the same genus indicates that this genotypic differentiation reflects adaptation of these *Archaea* to specific constraints imposed by the particular ecological niches that they occupy (Widderich et al., 2016a; Qin et al., 2017; Simon et al., 2017; Ren et al., 2019). We found in renewed database searches 30 archaeal genome sequences that contained *ectABC(D)* gene clusters. An alignment of these 30 archaeal EctB proteins with the bacterial (*Pl*)EctB protein as the search query yields an amino acid sequence identity of about 50%.

We found that the 13 amino acid residues in the crystal structure of the *A. aurescens* GABA-TA enzyme implicates in PLP and substrate binding (Figures 4C,E) are conserved in the (*Pl*)EctB protein as reveled by our *in silico* model (Figures 4D,F) and amino acid sequence alignments of 430 EctB-type proteins (Supplementary Figure S6). These apparently functionally important residues are either fully conserved (11 out of 13) in EctB-type proteins from *Bacteria* and *Archaea* or are conservatively replaced by functionally related amino acids (2 out of 13).

### The (*Pl*)EctB Enzyme Possess a Residual GABA-TA Activity

**FIGURE 5:**
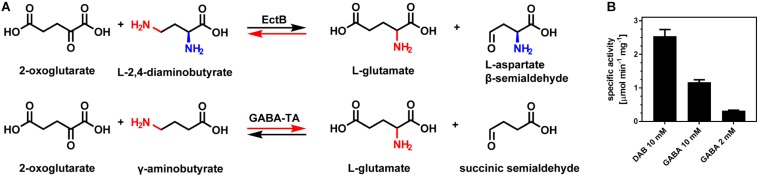
EctB and GABA-TAs catalyze highly similar reactions. **(A)** In its “reverse reaction,” EctB catalyzes the conversion of L-2,4-diaminobutyrate (DAB) and 2-oxoglutarate into L-glutamate and L-aspartate-β-semialdehyde (L-ASA), which is highly reminiscent to the forward reaction catalyzed by GABA-TAs. In their forward reaction, GABA-TAs catalyze the conversion of 2-oxoglutarate and γ-aminobutyrate (GABA) into L-glutamate and succinic semialdehyde. DAB and GABA, and their respective aldehydes, differ in an amino moiety (blue) only present in DAB or its aldehyde. In contrast to the amino group being transferred (red), this additional amino moiety is not involved in the EctB-mediated enzyme reaction. The red arrows indicate the forward reaction of EctB-type aminotransferases and GABA-TAs. **(B)** (*Pl*)EctB also catalyzes a GABA-TA-type reaction. Activities of (*Pl*)EctB for its reverse reaction with the substrates DAB and 2-oxoglutarate, and its reaction with the non-natural substrate GABA and 2-oxoglutarate were determined. In both cases, the formation of L-glutamate was used as the read-out for enzyme activity. The assays were performed using the optimal conditions of the (*Pl*)EctB enzyme for its reverse reaction. Two different concentrations of GABA were used. The error bars represent the standard deviation calculated from two technical and two biological replicates.

The GABA-TAs from *A. aurescens* and *E. coli* are the most closely related enzymes to EctB with respect to the catalyzed enzymatic reaction and quaternary assembly (Liu et al., 2004, 2005; Bruce et al., 2012). As TAs (aminotransferases) generally catalyze the interconversion of amino acids and keto acids (Steffen-Munsberg et al., 2015), GABA-TAs specifically convert GABA into succinic semialdehyde, while the amino group is transferred to 2-oxogluterate yielding L-glutamate (Figure 5A). Essentially all amino acid residues required for PLP binding to GABA-TAs (Liu et al., 2004, 2005; Bruce et al., 2012) are also conserved in EctB enzymes as shown by our *in silico* analysis (Figures 4C,D and Supplementary Figure S5). The substrates and products of the EctB “reverse reaction” and those of the forward reaction of GABA-TAs are strikingly similar (Figure 5A). A significant difference between the otherwise highly equivalent EctB- and GABA-TA-catalyzed reactions is the presence of one additional amino group at DAB or L-ASA (Figure 5A). However, this amino group is not relevant for the transamination reaction catalyzed by EctB.

As a mark of their evolutionary history, many extant enzymes have retained a certain degree of substrate ambiguity, thereby generating a metabolic pattern that is often addressed as underground metabolism (Jensen, 1976; D’Ari and Casadesus, 1998; Michael, 2017). Given the similarities between the EctB and GABA-TAs catalyzed reactions (Figure 5A) and the predicted structural relatedness of their active sites (Figures 4C–F), we wondered if the (*Pl*)EctB protein could use GABA as one of its substrates for the reverse reaction. This was indeed the case, and as expected for a side-reaction, use of GABA by the (*Pl)*EctB enzyme is inefficient (Figure 5B).

### *P. lautus* Possesses an Unusual Arrangement of Genes for Ectoine Import and Biosynthesis

**FIGURE 6:**
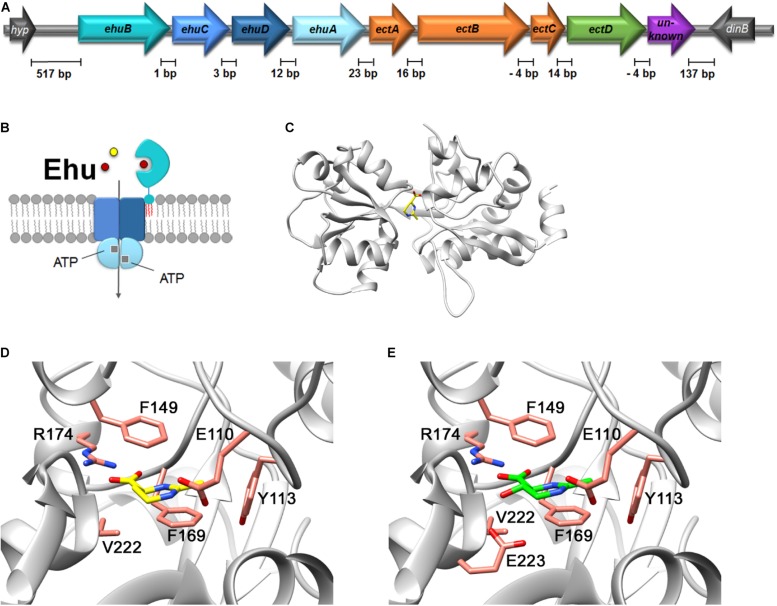
*In silico* analysis of the Ehu-like ABC-type transport system of *P. lautus* and its coding genes. **(A)** Genetic organization of the *ehu-ect* cluster (*ehuB*-*ehuC-ehuD-ehuA-ectA-ectB-ectC-ectD*) from *P. lautus*. **(B)** Schematic representation of the putative subunit composition of the Ehu ABC transport system from *P. lautus*. The extracellular substrate binding protein (*Pl*)EhuB of the Ehu ABC system is anchored to the outer face of the cytoplasmic membrane via a lipid modification (shown in red) at its amino-terminal Cys residue. **(C)** SWISS-MODEL of the (*Pl*)EhuB substrate-binding protein containing the ligand ectoine. The crystal structure of ectoine-bound *S. meliloti* EhuB protein (PDB-ID: 2Q88) served as template for modeling (Hanekop et al., 2007). The predicted architecture of the (*Pl*)EhuB substrate-binding site is shown for **(D)** ectoine and **(E)** 5-hydoxyectoine, with the latter structure modeled using the *S. meliloti* EhuB protein in complex with 5-hydroxyectoine (PDB-ID: 2Q89) as the template (Hanekop et al., 2007).

Often ectoine/hydroxyectoine biosynthetic gene clusters contain other genes involved in either the transcriptional regulation (*ectR*) of the *ect* operon, the provision of the precursor L-ASA (*ask_ect*), or sometimes, even a gene for a mechanosensitive channel (*mscS*) (Mustakhimov et al., 2010; Reshetnikov et al., 2011; Widderich et al., 2016a; Czech et al., 2018a). *P. lautus* possesses the canonical *ectABCD* ectoine/hydroxyectoine biosynthetic gene cluster (Figure 6A), but lacks the mentioned *ectR*, *ask_ect*, or *mscS* genes.

However, we found four genes encoding a binding-protein-dependent ABC transporter located upstream of the *P. lautus ectABCD* genes (Figure 6A). A closer analysis of these genes revealed that the encoded proteins are related to those of the functionally characterized ectoine/hydroxyectoine ABC-type uptake system EhuABCD from *S. meliloti* (Ehu: ectoine-hydroxyectoine-uptake) (Jebbar et al., 2005; Hanekop et al., 2007). The degree of amino acid identity between the components of the *S. meliloti* EhuABCD transport system and that of *P. lautus* were as follows: 60% for the nucleotide-binding domain EhuA (ATPase), 46% for the trans-membrane domain EhuC, 53% for the trans-membrane domain EhuD, and 35% for the substrate-binding protein EhuB. Therefore, we decided to retain the nomenclature of the *S. meliloti* Ehu system for the corresponding genes in *P. lautus*; we note however, that the genetic arrangement of the *ehu* gene clusters differs between these two microorganisms [*ehuABCD*/*ehuBCDA*] (Supplementary Figure S7A). The distances of individual genes within the *ect* and *ehu* gene clusters range between 1 and 16 base pairs. One can thus safely assume that they are co-transcribed. Notably, the distance between the *ehu* and *ect* gene clusters is only 23 base pairs, suggesting co-transcription of these two gene clusters as well (Figure 6A).

The substrate binding proteins operating in conjunction with microbial ABC transporters are located in the periplasm of Gram-negative bacteria (Davidson et al., 2008; Berntsson et al., 2010). Since Gram-positive bacteria lack an outer membrane, the corresponding proteins are either fused to a trans-membrane domain, or are tethered to the outer face of the cytoplasmic membrane via a lipid modification of a Cys residue at their mature amino-terminus (van der Heide and Poolman, 2002; Nguyen and Götz, 2016). The EhuB protein of the Gram-negative bacterium *S. meliloti* is located in the periplasm (Jebbar et al., 2005; Hanekop et al., 2007), while the corresponding protein from the Gram-positive bacterium *P. lautus* is a predicted lipoprotein (Nguyen and Götz, 2016; Figure 6B). Crystal structures of the *S. meliloti* EhuB protein in complex with either ectoine or hydroxyectoine have been reported (PDB-IDs: 2Q88 and 2Q89, respectively) (Hanekop et al., 2007). These two crystal structures allowed us to create an *in silico* model of the *P. lautus* EhuB protein (Figure 6C) with the aid of the SWISS MODEL server (Waterhouse et al., 2018). This model shows that all residues involved in ectoine and hydroxyectoine binding by the *S. meliloti* EhuB protein (Figures 6D,E) are conserved in the (*Pl*)EhuB protein (Supplementary Figure S8). These findings further support our idea that the *ehuBCDA* genes in *P. lautus* encode indeed an uptake system for ectoines.

To further support our modeling and amino acid sequence alignment approaches suggesting that (*Pl*)EhuB was a substrate binding protein recognizing ectoines, we subjected the (*Pl*)EhuB model to a molecular docking experiment using AutoDock Vina (Trott and Olson, 2010) and with ectoine as the input molecule. The position of ectoine in the (*Pl*)EhuB docking model (Supplementary Figure S9) is very similar to the orientation of the ectoine molecule found in the crystal structure of the EhuB:ectoine complex from *S. meliloti* (Hanekop et al., 2007). The docked ectoine molecule in (*Pl*)EhuB is coordinated by a similar set of amino acids found in the experimentally determined (*Sm*)EhuB:ectoine complex: Arg-174 coordinates the carboxyl group of ectoine and two phenylalanines (Phe^149^ and Phe^169^) and a tyrosine residue (Tyr^113^) provide further stabilizing contacts (Supplementary Figure S9) via π–π interactions as detailed in the previous report on the EhuB crystal structure by Hanekop et al. (2007). Consequently, the (*Pl*)EhuB:ectoine model derived by a molecular docking approach (Trott and Olson, 2010) is fully in agreement with the (*Pl*)EhuB:ectoine or hydroxyectoine models (Figures 6D,E) obtained with the algorithm(s) used by the SWISS-MODEL server (Waterhouse et al., 2018).

Having established that (*Pl*)EhuB is in all likelihood a substrate binding protein for ectoines, we wondered whether the *ehu-ect* gene cluster was a unique feature of the *P. lautus* strain Y4.12MC10 (Mead et al., 2012), or whether was a conserved feature in other members of the physiologically diverse *Paenibacillus* genus as well (Dsouza et al., 2014; Grady et al., 2016). We therefore queried the IMG/M database (Chen et al., 2019) for genome sequences of Paenibacilli. At the time of the search (04 June 2019), 288 sequences were represented; 16 genome sequences were finished, 85 genome sequences had been deposited as “permanent drafts,” and 187 draft sequences were available. Using the (*Pl*)EctC protein as the search query, we found 41 EctC orthologs, and in each case, the corresponding gene was part of an *ectABC(D)* gene cluster [among these were 38 *ectABCD* gene clusters]. Having this information at hand, we inspected the genome context of the 41 *ect* gene clusters using annotation tools provided by the IMG/M web server (Chen et al., 2019). Notably, in 37 out of the 41 inspected *ect* genome neighborhood, we found *ehu* gene clusters in a genetic configuration (*ehuBCDA*) identical to that of *P. lautus* strain Y4.12MC10 (Figure 6A). In a further step of our analysis, we aligned the amino acid sequences of the EhuB-type substrate-binding proteins and found a sequence identity between these proteins ranging between 99 (for *Paenibacillus* sp. FSL_H8-457) and 65% (for *Paenibacillus senegalensis* JC6). Six of the seven amino acids that are predicted to form the EhuB ectoine/hydroxyectoine ligand-binding pocket (Figures 6D,E) are strictly conserved [corresponding the Glu^110^, Tyr^113^, Phe^149^, Phe^169^, Arg^174^, Glu^223^ of (*Pl*)EctB] in the 37 proteins, while Val^222^ is in a number of cases conservatively replaced by either an Ile or an Ala residue. An abbreviated alignment of 10 EhuB-type ligand-binding proteins is shown in Supplementary Figure S8 that shows the conservation of those residues implicated in ectoine/hydroxyectoine binding (Hanekop et al., 2007). Collectively, our modeling studies and phylogenomic analysis suggest that all of these ligand-binding proteins recognize ectoines as their substrates. Hence, an *ehuBCDA-ectABCD* operon-like structure is an evolutionarily conserved arrangement in many Paenibacilli.

## Discussion

### Basic Properties of the (*Pl*)EctB L-2,4-Diaminobutyrate Transaminase

The EctB L-2,4-diaminobutyrate TA (aminotransferase) is the first enzyme of the ectoine biosynthetic route (Peters et al., 1990; Ono et al., 1999; Pastor et al., 2010; Reshetnikov et al., 2011; Czech et al., 2018a) and converts L-glutamate and L-ASA into 2-oxoglutarate and DAB (Figure 1). Only two EctB enzymes have previously been characterized biochemically and only to some extent (Ono et al., 1999; Reshetnikov et al., 2006; Chen et al., 2015). In continuation of our efforts to characterize the ectoine biosynthetic enzymes (so-far the ectoine synthase EctC and the ectoine hydroxylase EctD) (Höppner et al., 2014; Czech et al., 2019), we focused here on the L-2,4-diaminobutyrate TA EctB and we used the EctB protein from the thermotolerant Gram-positive bacterium *P. lautus* (*Pl*) for these studies. (*Pl*)EctB is a highly resilient enzyme with respect to elevated temperatures, salt, and pH (Figures 3A–D). Given the interest in TAs for practical purposes (Steffen-Munsberg et al., 2015), the robust *P. lautus* EctB protein might provide opportunities to exploit this enzyme for biotechnological applications.

(*Pl*)EctB differs from its *H. elongata* counterpart (Ono et al., 1999), as it does not need higher concentrations of K^+^ for its enzyme activity or stability (Figure 3C). The EctB proteins of *H. elongata* and *M. alcaliphilum* were previously described to form hexamers based on SEC analysis calibrated with globular reference proteins (Ono et al., 1999; Reshetnikov et al., 2011). However, our analysis of the *P. lautus* EctB protein analyzed by MALS-RI shows a tetramer (Figure 2B). These differences might be due to species-specific variations in the quaternary assembly of EctB proteins, but alternatively, might represent an analytical problem. The estimation of molecular mass by SEC calibrated with globular standards can be biased by protein shape. In contrast, MALS-RI measurements provide the absolute molecular mass, because data obtained by this analytical technique are not influenced by the shape of a given protein (Oliva et al., 2004; Sahin and Roberts, 2012). EctB proteins are closely related to the GABA-TAs from *A. aurescens* and *E. coli* (Liu et al., 2004, 2005; Bruce et al., 2012; Supplementary Figure S4), whose crystal structures display a homo-tetrameric arrangement (Figure 5A). Therefore, we suggest that EctB proteins are, in all likelihood, in general homo-tetramers in solution.

### Mechanistic Implications for EctB as a PLP-Dependent GABA-Like Transaminase

Transaminases (aminotransferases) are PLP-dependent enzymes, and when purified, frequently exhibit a yellow color that can be exploited for photospectrometric analysis (Steffen-Munsberg et al., 2015). Solutions of the purified recombinant (*Pl*)EctB protein did not exhibit this yellow color; hence, (*Pl*)EctB is apparently produced in *E. coli* as an apo-protein that was enzymatically inactive (Figure 2C). However, enzyme activity could be recovered by adding PLP to the assay solutions (Figure 2C). Lys^274^ was identified through our modeling (Figure 4D and Supplementary Figure S4C) and site-directed mutagenesis experiments (Figure 2E) as the residue to which PLP can be covalently attached in (*Pl*)EctB via its aldehydic C-atom. Not surprisingly, this residue is strictly conserved in an amino acid sequence alignment of 430 *bona-fide* EctB proteins that comprises representatives from bacterial and archaeal phyla (Czech et al., 2018a) (see Supplementary Figure S6 for an abbreviated sequence alignment of 10 bacterial EctB proteins).

Caused by the limited commercial availability of the authentic EctB substrate L-ASA (Figure 1), others and we have used the “reverse reaction” to assess the kinetic properties of EctB (Ono et al., 1999; Reshetnikov et al., 2006, 2011; Chen et al., 2015; Figures 3E,F). Our biochemical analysis shows that (*Pl*)EctB efficiently catalyzes in its “reverse reaction” the transamination of the 2’ amino group of DAB onto 2-oxoglutarate in order to form glutamate and L-ASA (Figures 1, 5A and Table 1). Using custom synthesized L-ASA, Ono et al. (1999) previously showed that *H. elongata* EctB can readily catalyze the forward reaction, a reaction that yields DAB as the first intermediate in ectoine biosynthesis (Figure 1). Viewing the ectoine/5-hydroxyectoine biosynthetic route as a whole (Peters et al., 1990; Ono et al., 1999; Pastor et al., 2010; Reshetnikov et al., 2011; Kunte et al., 2014; Czech et al., 2018a), the 2-oxoglutarate formed as the second reaction product of the EctB enzyme in its forward reaction (Figure 1) can subsequently be used by the EctD ectoine hydroxylase as a substrate (Figures 1, 5A). EctD is a member of the non-heme-containing iron(II) and 2-oxoglutarate-dependent dioxygenases (Höppner et al., 2014; Widderich et al., 2014b, 2016a).

Collectively, the kinetic analysis of the *H. elongata* (Ono et al., 1999) and *P. lautus* (this study) EctB enzymes shows that EctB is able to catalyze both opposing transamination reactions with similar catalytic efficiencies, albeit the forward reaction seems preferred with respect to the *V*_*max*_ value by about four-fold as assessed with the *H. elongata* enzyme (Ono et al., 1999; Table 1). Therefore, the cellular concentrations of available substrates should primarily dictate the catalytic directionality of the EctB enzyme. However, the onset of enhanced *ectABC(D)* transcription under osmotic stress conditions (Kuhlmann and Bremer, 2002; Pastor et al., 2010; Czech et al., 2018a, b; Stiller et al., 2018) will drive the flow of L-ASA into the ectoine biosynthetic route (Figure 1). This is probably the reason why a substantial number of ectoine/hydroxyectoine-producing microorganisms co-express the *ectABC(D*) biosynthetic genes with the gene for a specialized aspartokinase (Ask_ect) (Reshetnikov et al., 2006; Stöveken et al., 2011; Czech et al., 2018a) in order to avoid the build-up of a metabolic bottleneck during enhanced production of ectoines (Bestvater et al., 2008; Lo et al., 2009; Kunte et al., 2014).

The crystal structure of the *A. aurescens* GABA-TA bound to its PLP-GABA adduct (the external aldemine) (PDB-ID: 4ATQ) shows that the carboxyl group of GABA is tightly coordinated by the side chain of an arginine residue strictly conserved throughout this enzyme family (Figure 4E; Bruce et al., 2012). Each active site of GABA-TA is complemented by a loop of the adjacent subunit in the context of the homo-tetrameric assembly (see for instance the loop marked in gray stemming from the adjacent monomeric subunit in Figures 4A,E; Bruce et al., 2012). A structural comparison of GABA-TA with our model of (*Pl)*EctB (Figures 4A,B) shows that the DABA-coordinating arginine is replaced by a conserved methionine in EctB enzymes (Figures 4D,F and Supplementary Figure S4C). Taken together, our analysis shows that the active site of EctB enzymes slightly differs from their otherwise highly conserved GABA-TA counterparts in order to accommodate the extra amino moiety present in the substrate (L-ASA) for its forward reaction, and the generated reaction product DAB (Figure 5A).

Our analysis suggests that the PLP-dependent EctB aminotransferase and GABA-TAs conduct similar types of enzyme reactions as they exhibit closely related chemical structures of their substrates and products. However, the directionality of the physiology most relevant catalyzed reaction of these two enzymes is just the opposite (Figure 5A). It is well known, that in addition to their major substrate, enzymes are frequently promiscuous with respect to their use of chemically related compounds (Jensen, 1976; D’Ari and Casadesus, 1998; Michael, 2017). Given the chemically related enzyme reactions catalyzed by GABA-TAs and EctB (Figure 5A) and the predicted structural relatedness of the active sites of these two types of enzymes (Figures 4C–F), we wondered if the (*Pl*)EctB protein could use GABA as one of its substrates for the reverse reaction. Indeed, (*Pl*)EctB can use GABA as a substrate, albeit, and as expected, inefficiently (Figure 5B). This finding buttresses the structural (Figure 4 and Supplementary Figure S4) and enzymological relatedness (Figures 5A,B) of EctB-type aminotransferases and GABA-TAs that we propose in this manuscript.

**FIGURE 7:**
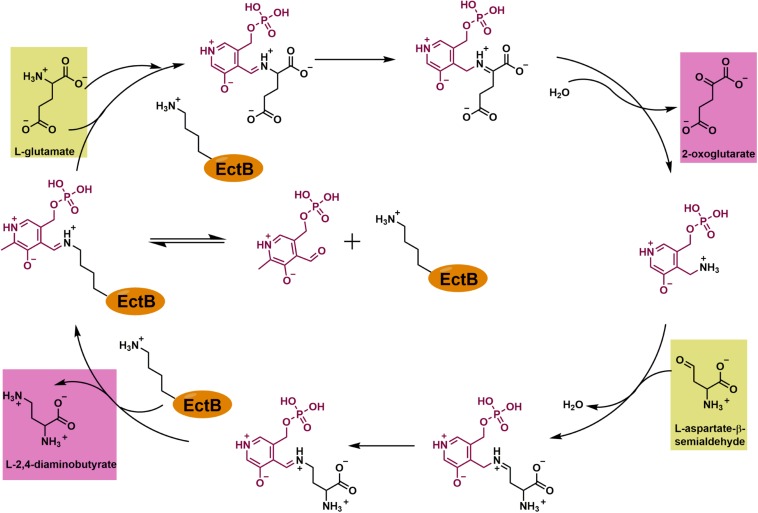
Enzyme mechanism of EctB. In the resting state of the EctB enzyme, the PLP cofactor is bound to Lys^274^ as a Schiff-base (the “internal aldemine”). EctB catalyzes two half-reactions: In the first one, the donor of the amino-group, L-glutamate, is transferred onto the Lys^274^-bound PLP molecule, thereby forming a PLP-L-glutamate adduct (the “external aldemine”), which then further reacts to release 2-oxoglutarate and the modified co-factor pyrodoxamine-5′-monophosphate (PMP). In the second half-reaction, PMP reacts with the second substrate, L-ASA, that serves as the amino-group acceptor molecule. In this sequence of events, the PLP-L-ASA adduct reacts with the now free side chain of Lys^274^, thereby releasing DAB and reforming the Lys^274^-PLP “internal aldemine” again. The reaction scheme proposed here for EctB-type enzymes corresponds closely to PLP-dependent transaminases in general (Steffen-Munsberg et al., 2015) and GABA-TAs in particular (Liu et al., 2004, 2005; Bruce et al., 2012).

Collectively, our data allow us to make a proposal for the enzyme reaction catalyzed by EctB during the synthesis of ectoine (Figure 7). The suggested individual steps carried out during catalysis by EctB closely follow those of GABA-TAs, enzymes that have been studied biochemically and structurally in detail (Liu et al., 2004, 2005; Bruce et al., 2012; Steffen-Munsberg et al., 2015). In the resting, the internal aldemine stage of the EctB enzyme, PLP is covalently attached to Lys^274^. Reaction of the substrate L-glutamate with the Lys^274^-bound PLP leads to the formation of a PLP-L-glutamate adduct (the external aldemine) that is no longer covalently attached to the side-chain of Lys^274^; concomitantly the EctB reaction product 2-oxoglutarate is released. The modified PLP molecule (carrying now an NH_3_ group that is derived from the substrate L-glutamate) (Figure 7) can then react with the second substrate of EctB, L-ASA, to form a PLP-DAB adduct. In a further reaction step, the second reaction product of EctB, DAB, is released and the resting stage of the enzyme is re-formed via the covalent attachment of PLP to the side-chain of Lys^274^, yielding again the internal aldemine (Figure 7).

### The *ehu-ect* Gene Cluster in *Paenibacilli*: Ecophysiological Considerations

We discovered an unusual genetic organization of the *ectABCD* genes in the genome sequence of the *P. lautus* strain Y412MC10 (Mead et al., 2012), as they are positioned next to a gene cluster (*ehuBCDA*) encoding a binding-protein-dependent ABC transport system (Figures 6A,B). Notably, all residues required for ligand binding by the *P. lautus* EhuB substrate binding protein are conserved, as revealed by a comparison with the EhuB counterpart from *S. meliloti*, of which crystal structures with either ectoine or 5-hydroxyectoine as their ligands are available (Figures 5D,E; Hanekop et al., 2007). Together, our modeling and docking studies with (*Pl*)EhuB leave little doubt that the *P. lautus* Ehu ABC transporter (Figure 5B) serves as an uptake system for ectoine and 5-hydroxyectoine.

The transcription of the genes for the Ehu ABC transporter from the plant roots-associated bacterium *S. meliloti* is inducible by ectoine along with juxtapositioned genes for the catabolism of this compound (Jebbar et al., 2005). In contrast, the *P. lautus* Ehu transport system must be primarily involved in osmostress adjustment as the genome sequence of *P. lautus* (Mead et al., 2012) lacks ectoine catabolic gene clusters (Jebbar et al., 2005; Schwibbert et al., 2011; Schulz et al., 2017). The distances between the individual genes in the *ehu* and *ect* loci, and the distance between the two gene clusters (23 bp) are such that one can reasonably assume that the *ehu-ect* genes form an operon in *P. lautus* (Figure 6A). This genetic arrangement is found in 37 of the 41 Paenibacilli that possess *ect* genes among the 288 inspected genome sequences of members of the *Paenibacillus* genus. We know of only one published example where an *ect-ehu* gene cluster has been described, namely, in the Nitrospina sp. SCGC_AAA799_C22 isolate, a bacterium that lives in the poly-extreme interfaces of Red Sea brines (Ngugi et al., 2016). We now found four additional examples of *ect-ehu* gene clusters in recently reported genome sequences of members of this genus (Supplementary Figure S7B).

Synthesis and import of ectoines provides not only a considerable degree of osmotic stress protection, but also ameliorates high temperature-induced cellular stress (Garcia-Estepa et al., 2006; Bursy et al., 2008). As the *P. lautus* strain Y412MC10 was isolated from the effluent of a hot spring in the Yellowstone National Park (United States) (Mead et al., 2012), synthesis and import of ectoines might be an adaptive trait to cope with the challenges posed by this habitat with its elevated temperatures. *P. lautus* can grow at 50°C under laboratory conditions. However, annotation of its genome sequence indicates that it is in all likelihood an intestinal bacterium that was probably brought inadvertently into the hot spring by fecal material deposited by mammals bathing in the warm water (e.g., by bison) (Mead et al., 2012). Interestingly, the *P. lautus* genome shares a high degree of synteny and homology to the human isolates *Paenibacillus vortex*_V453 and *Paenibacillus* sp. HGF5, the latter of which is a microorganism from the Human Microbiome Project Reference Genomes (Human Microbiome Jumpstart Reference Strains Consortium et al., 2010). Both Paenibacilli possess the same *ehu-ect* gene cluster that we describe here for *P. lautus* Y412MC10 (Figure 6A), raising the question whether the accumulation of ectoines plays a protective role in gut microbiomes?

In those Paenibacilli that possess *ehu-ect* gene clusters, ectoine uptake and synthesis seems to be tightly coordinated. Transport systems for compatible solutes serve to scavenge these stress protectants from scarce environmental sources (Kempf and Bremer, 1998; Bremer and Krämer, 2000; Wood et al., 2001). However, they frequently also function as recycling systems for newly synthesized compatible solutes that are either actively excreted or are leaking through the cytoplasmic membrane of the producer cells, possibly in an effort to fine tune turgor (Lamark et al., 1992; Hagemann et al., 1997; Grammann et al., 2002; Börngen et al., 2010; Hoffmann et al., 2012). Indeed, the genetic disruption of the high-affinity ectoine/5-hydroxyectoine TRAP-type TeaABC transporter from *H. elongata* created an overproducing strain for ectoine and leads to the accumulation of considerable amounts of ectoine in the growth medium (Grammann et al., 2002). As microorganisms frequently live in their natural habitats in mono- or multi-specific assemblages and biofilms, secreted/released ectoines might become a public good from which those bacteria that possess the appropriate uptake system will particularly benefit (Kapfhammer et al., 2005; Ren et al., 2017; Bremer and Krämer, 2019).

## Data Availability Statement

All datasets generated for this study are included in the article/Supplementary Material.

## Author Contributions

EB conceived and directed the study. AR, C-NM, LC, KG, AH, and SS performed the experiments, analyzed the data, and interpreted the results. EB, GB, and TE discussed the data. AR, GB, and EB wrote the manuscript. All authors commented on the manuscript.

## Conflict of Interest

The authors declare that the research was conducted in the absence of any commercial or financial relationships that could be construed as a potential conflict of interest.

## Footnotes

1 https://www.ncbi.nlm.nih.gov/nuccore/MK682511.1

2 https://www.iba-lifesciences.com/cloning-entry-fusion-vectors.html

3 https://www.iba-lifesciences.com/cloning-ecoli-vectors.html

4 https://img.jgi.doe.gov/

5 http://mafft.cbrc.jp/alignment/server/

6 https://swissmodel.expasy.org/

7 https://www.cgl.ucsf.edu/chimera/

8 https://pymol.org/2/

9 https://swissmodel.expasy.org/

10 http://www.sbg.bio.ic.ac.uk/~phyre2/html/page.cgi?id=index
