# Supplementary material for: Biosynthesis of the Stress-Protectant and Chemical Chaperon Ectoine: Biochemistry of the Transaminase EctB
Source: Front Microbiol. 2019 Dec 10;10:2811. doi: 10.3389/fmicb.2019.02811 (PMC6915088; doi:10.3389/fmicb.2019.02811)
Supplement: Supplementary file 1 [file Data_Sheet_1.PDF]

Biosynthesis of the stress-protectant and chemical chaperon ectoine: biochemistry of the transaminase EctB

Alexandra A. Richter[1,2], Christopher-Nils Mais[2,3], Laura Czech[1,2], Kyra Geyer[4], Astrid Hoeppner[5] Sander H.J. Smits[5,6], Tobias J. Erb[2,4], Gert Bange[2,3], and Erhard Bremer[1,2]*

[1]Laboratory for Microbiology, Department of Biology, Philipps-University Marburg, Marburg, Germany
[2]SYNMIKRO Research Center, Philipps-University Marburg, Marburg, Germany
[3]Department of Chemistry, Philipps-University Marburg, Marburg, Germany
[4]Max-Planck-Insitute for Terrestrial Microbiology, Department of Biochemistry and Synthetic Metabolism, Marburg, Germany
[5]Center for Structural Studies, Heinrich-Heine University Düsseldorf, Düsseldorf, Germany
[6]Institute of Biochemistry, Heinrich-Heine University Düsseldorf, Düsseldorf, Germany

For correspondence during the reviewing and editorial process please contact:

Dr. Erhard Bremer, Philipps-University Marburg, Dept. of Biology, Laboratory for Microbiology, Karl-von-Frisch-Str. 8, D-35032 Marburg, Germany. Phone: (+49)-6421-2821529. Fax: (+49)-6421-2828979. E-Mail: bremer@staff.uni-marburg.de

____

*Correspondence:

Erhard Bremer: bremer@staff.uni-marburg.de

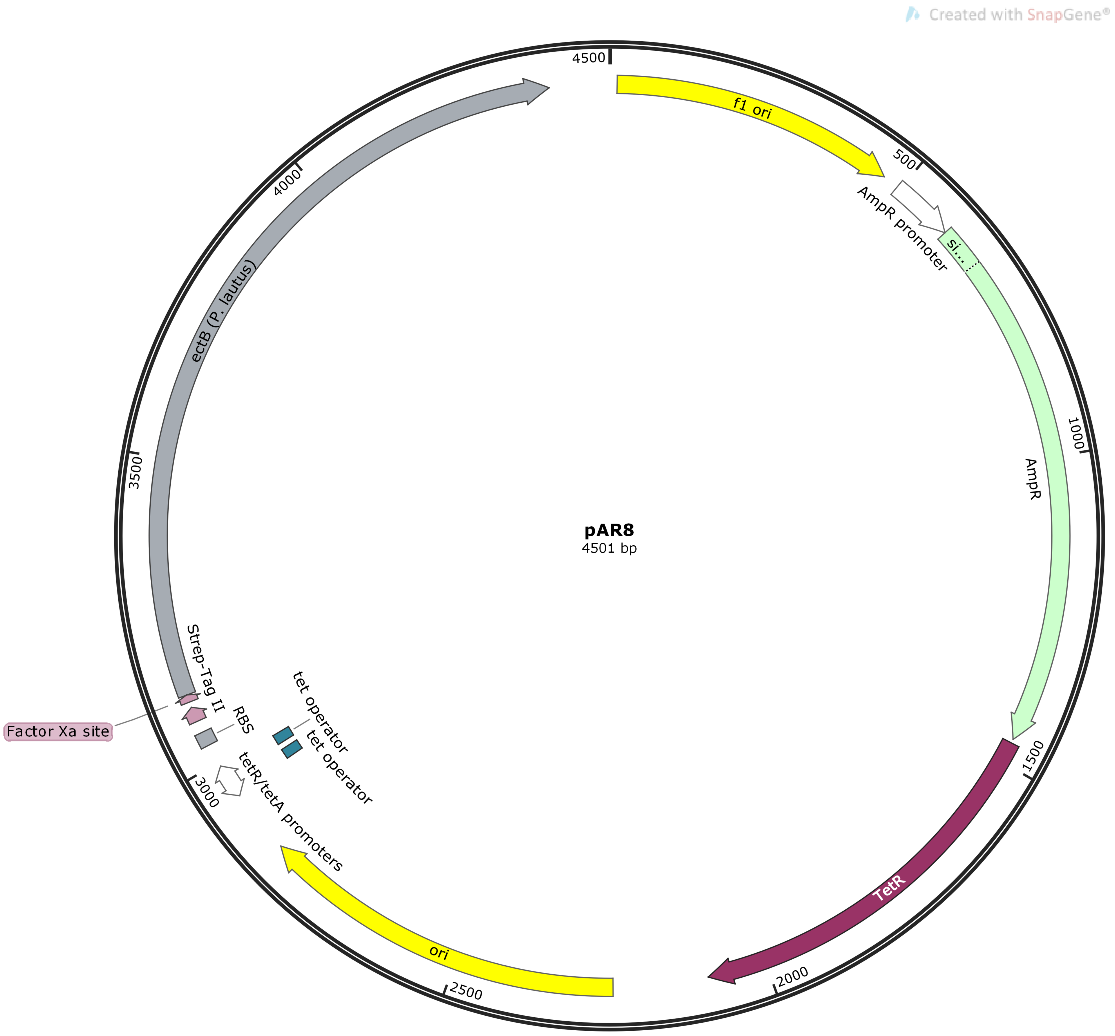

Supplementary Figure S1 │ Genetic structure of the (*Pl*)*ectB*-expression vector pAR8. This expression vector carries an ampicillin resistance gene (β-lactamase gene, including the sequence for the signal peptide guiding the beta-lactamase into the periplasm) to allow selection in the chosen expression organism. The plasmid backbone carries the *tet* promoter and the gene for the tetracycline repressor TetR controlling the transcriptional activity of the tet promoter in response to the synthetic inducer AHT. The recombinant (*Pl*)EctB protein is fused to an N-terminal *Strep*-Tag II via a Factor-Xa-site-containing linker sequence; it is expressed from the *tet* promoter under the control of a TetR repressor.

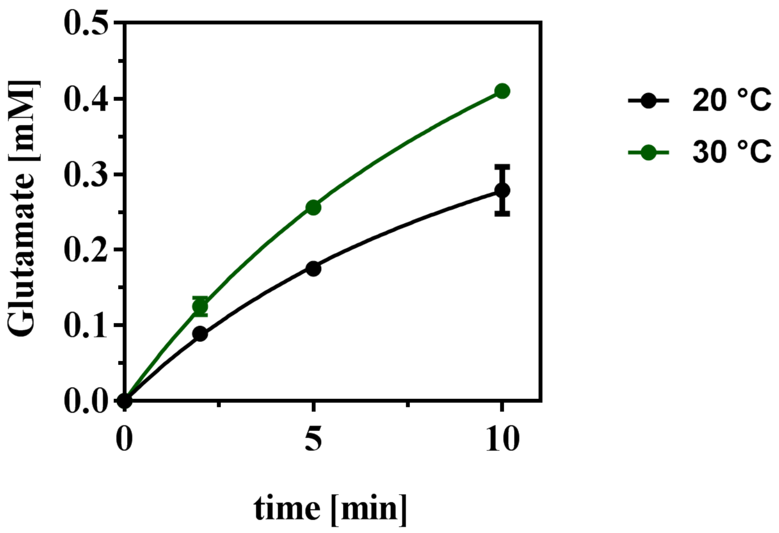

Supplementary Figure S2 │ Time dependent formation of L-glutamate during the (*Pl*)EctB reverse reaction. The observation of (Pl)EctB enzyme activity over a time scale of ten minutes shows the non-linear glutamate production by (*Pl*)EctB at later time points at two different assay temperatures. These assays were performed for the identification of the time range in which the (*Pl*)EctB catalyzed enzyme reaction proceeds linearly. This reaction time (1 min) was used for the determination of the kinetic parameters of the (*Pl*)EctB enzyme.

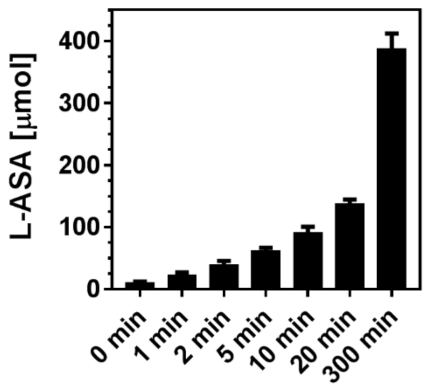

Supplementary Figure S3 │ Time course of L-ASA production by (*Pl*)EctB during its reverse reaction. Error bars are the standard deviation calculated from 2 technical and 2 biological replicates.

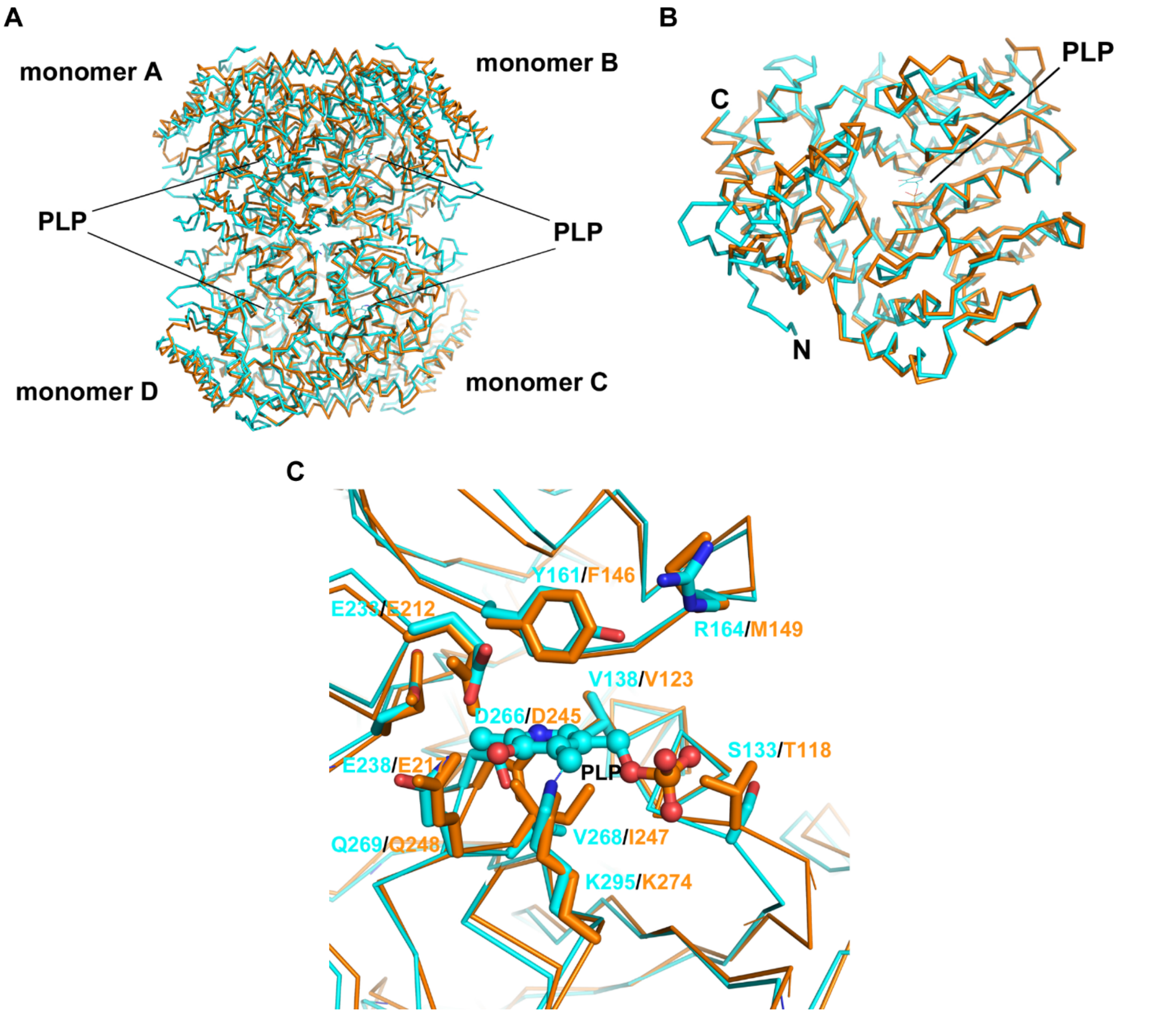

Supplementary Figure S4 │ Superimposition of the (*Pl*)EctB-Model and the (*Aa*)GABA-TA (PDB: 4ATP). (**A**) Superimposition of a tetrameric model (orange) of (*Pl*)EctB with the crystal structure (*Aa*)GABA-TA (PDB: 4ATP, blue). The root mean square deviation (RMSD) for each of the monomers in the quaternary assembly is 0.854 Å over 1852 atoms. (B) Superimposition of a monomeric model of (*Pl*)EctB (ornage) with the crystal structure of (*Aa*)GABA-TA (PDB: 4ATP, blue). **(C**) PLP-binding site of the (*Pl*)EctB-Model (green) and the (*Aa*)GABA-TA (blue, PDB: 4ATP).

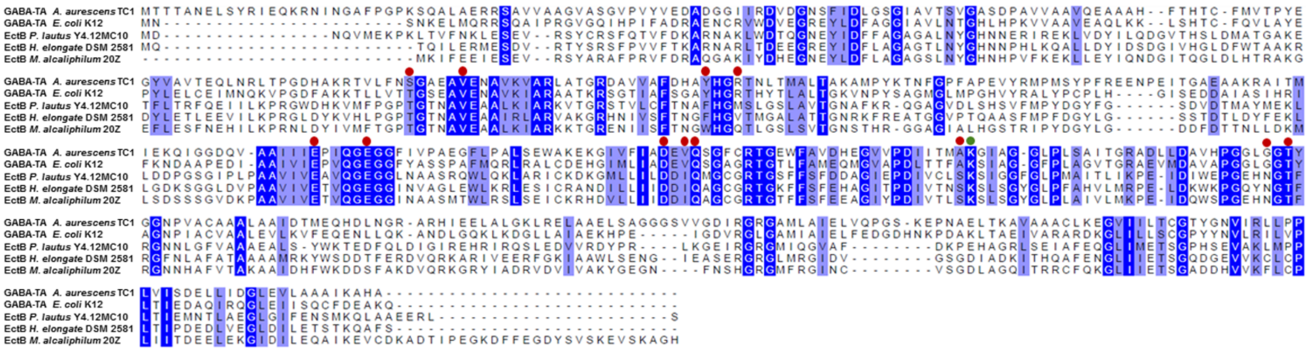

Supplementary Figure S5 │ Amino acid sequence alignment of different EctB and GABA-TA proteins. The alignment was generated with the MAFFT server (https://mafft.cbrc.jp/alignment/server/). Strictly conserved amino acids are shaded in blue. Red and green dots indicated the amino acids involved in ligand binding (red) and the PLP-binding residue (green) Lys^{274}.

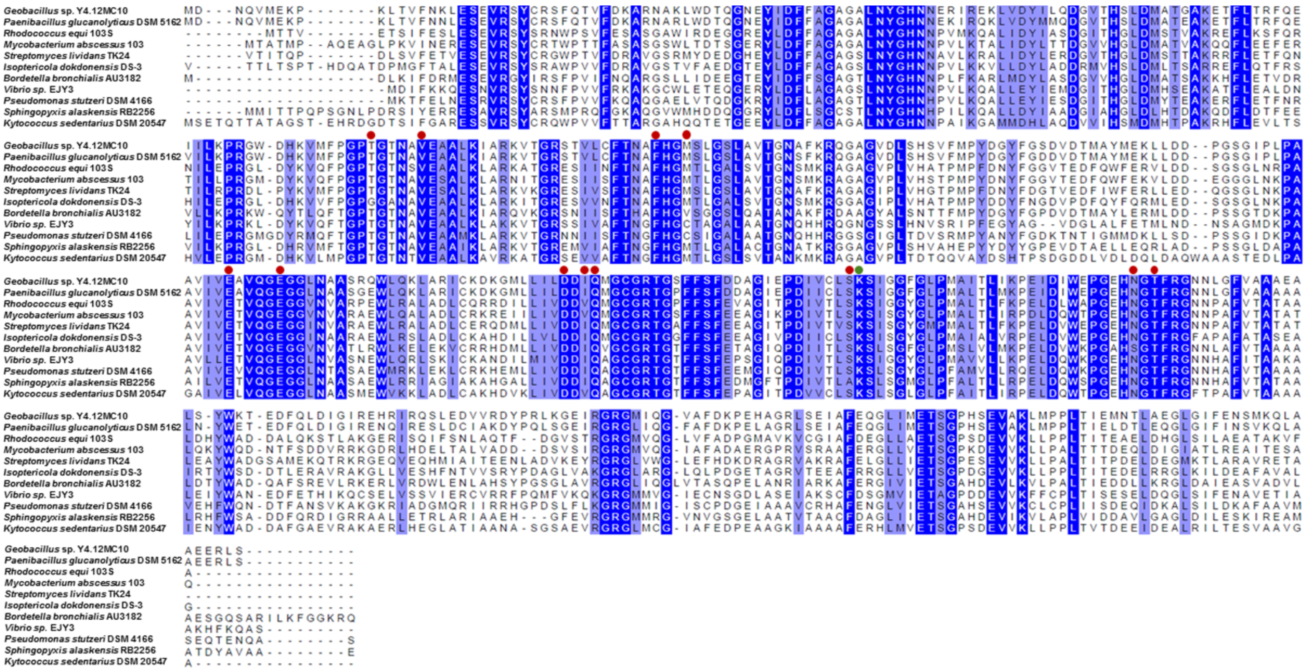

Supplementary Figure S6 | Amino acid sequence alignment of randomly selected EctB proteins. The alignment was generated with the MAFFT server (https://mafft.cbrc.jp/alignment/server/). Strictly conserved amino acids are shaded in blue. Red and green dots indicated the amino acids involved in ligand binding (red) and the PLP-binding residue (green) Lys^{274}.

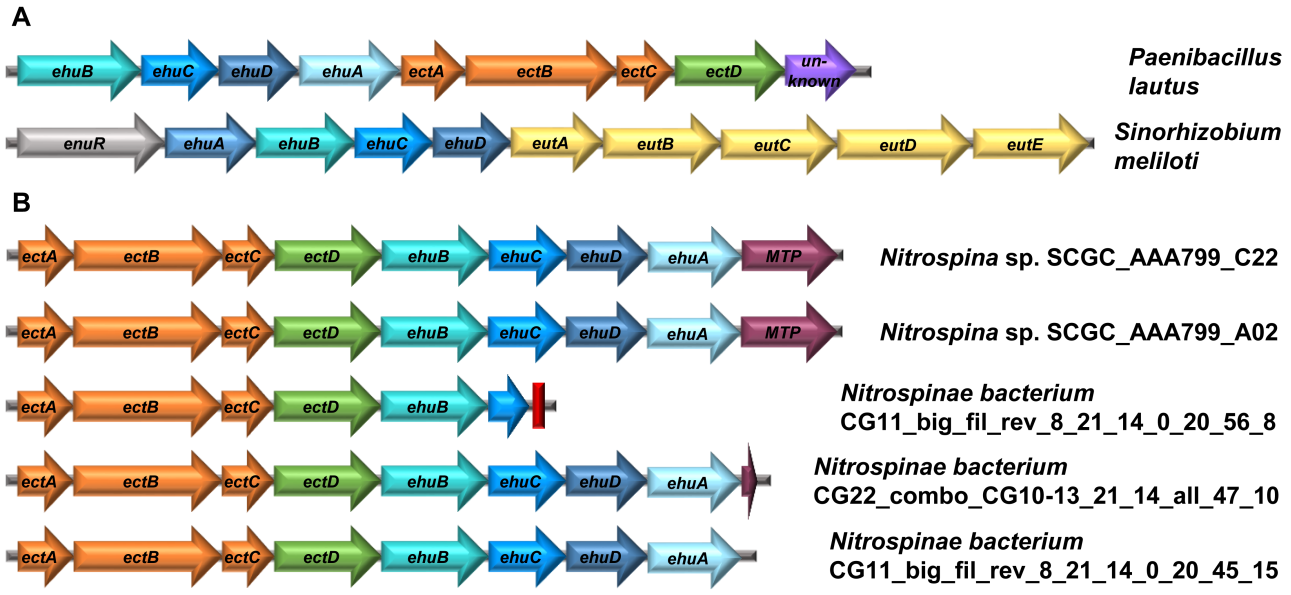

Supplementary Figure S7 │ Genetic organization of the of the *ehu-ect* cluster (*ehuB-ehuC-ehuD-ehuA-ectA-ectB-ectC*) from *P. lautus* and the *ehu*-cluster (*ehuA-ehuB-ehuC-ehuD*) from *S. meliloti*, neighbored with the gene encoding for the regulator EnuR and the *e*ctoine *u*tilization genes (*eutABCD*E) **(A)**. Those members of the *Nitrospinae* genus that possess ectoine/hydroxyectoine synthesis genes, also possess *ehu*-type transporter genes next to the ectoine/hydroxectoine biosynthetic gene cluster (*ectABCD-ehuBCDA*) **(B)**.

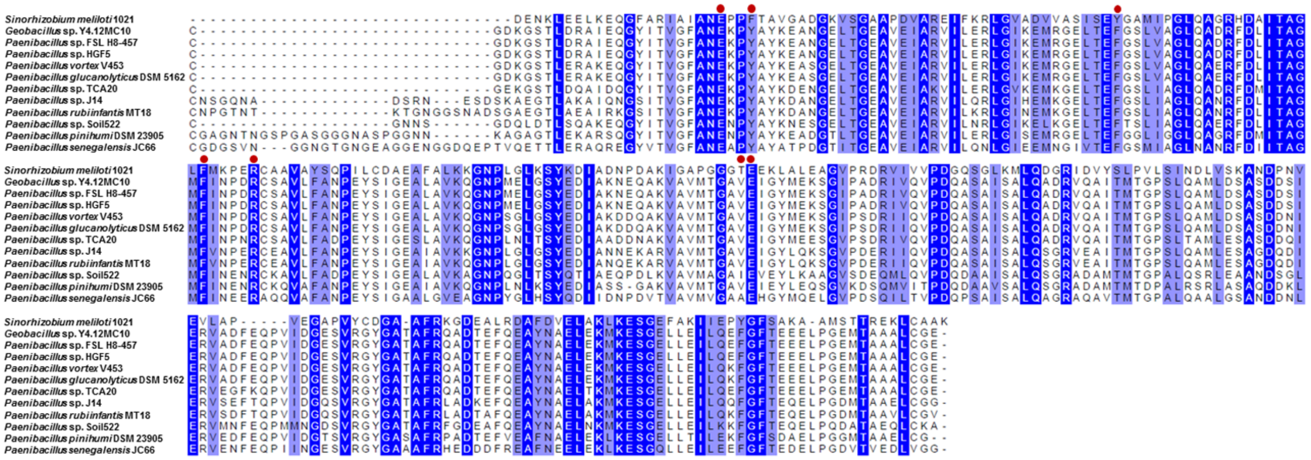

Supplementary Figure S8 │ Amino acid sequence alignment of 10 randomly chosen EhuB-type proteins. Sequences were randomly chosen from 37 *ehuB*-genes found in the genus *Paenibacillus*. The alignment was generated with the MAFFT server (https://mafft.cbrc.jp/alignment/server/). Strictly conserved amino acids are shaded in blue. Red dots indicate amino acids predicted to be involved in ligand binding (see **Figure 5** of main text).

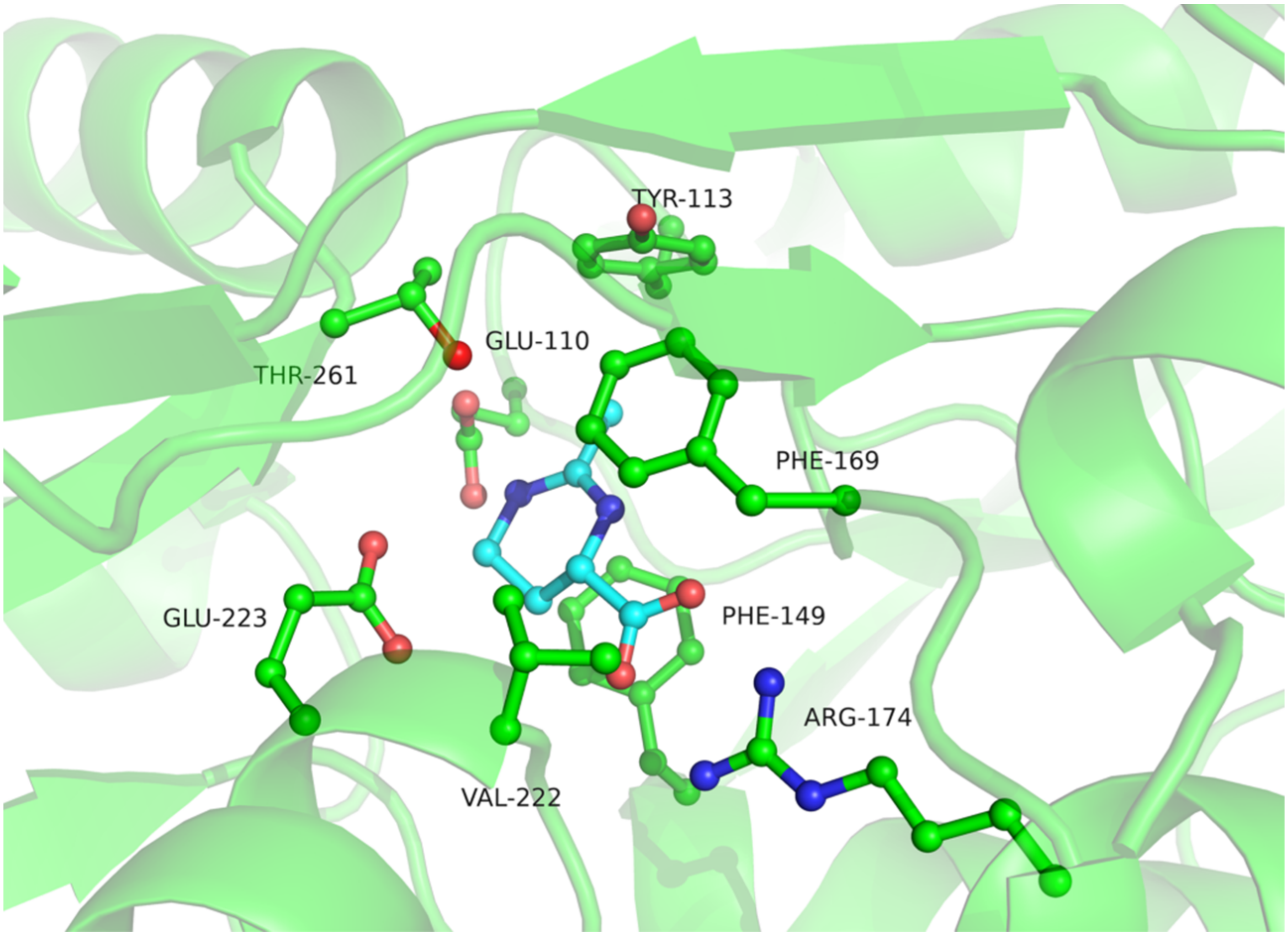

Supplementary Figure S9 | Architecture of the ectoine-binding site in the (*Pl*) EhuB substrate binding protein. The interactions of ectoine (shown in light blue sticks) with selected residues of the (*Pl*)EhuB protein are shown. The ectoine molecule is predominantly bound via electrostatic interactions between Arg[174] and the carboxyl-group of ectoine. A second set of interaction involves π-π between the three aromatic amino acid side chains of Tyr[113], Phe[169], and Phe[149] with the ring structure of ectoine. This figure was prepared by docking the ectoine ligand into the (*Pl*)EhuB *in silico* model build on the crystal structure of the periplasmic *S. meliloti* EhuB substrate binding protein in complex with ectoine (PDB-number 2Q88).

.

.

Supplementary Table S1 | Primers used in this study.

**Primer name**	**Primer sequence**
pLC47_for	CATAAGCTCTTCAATGGATAATC
pLC47_rev	CAGAAGCTCTTCACCCG
Strep_XA_EctB_Mut_R	cttcgatCATTCCGGATTTTTCGAATTG
Strep_XA_EctB_Mut_F2	gtcgtGATAATCAGGTGATGGAAAAAC
Q5_EctB_K274A_F	TTGTCTGAGCgcgAGCATTGGTG
Q5_EctB_K274_R	ACAATATCCGGTTCAATAC
Q5_EctB_K274H_F	TTGTCTGAGCcatAGCATTGGTG
Q5_EctB_K274R_F	TTGTCTGAGCcgtAGCATTGGTG
